# Efficient seeding for error-prone sequences with SubseqHash2

**DOI:** 10.1093/bioinformatics/btaf418

**Published:** 2025-07-24

**Authors:** Xiang Li, Ke Chen, Mingfu Shao

**Affiliations:** Department of Computer Science and Engineering, The Pennsylvania State University, Pennsylvania, PA 16802, United States; Department of Computer Science and Engineering, The Pennsylvania State University, Pennsylvania, PA 16802, United States; Department of Computer Science and Engineering, The Pennsylvania State University, Pennsylvania, PA 16802, United States; Huck Institutes of the Life Sciences, The Pennsylvania State University, Pennsylvania, PA 16802, United States

## Abstract

**Motivation:**

Seeding is an essential preparatory step for many fundamental computational tasks that require large-scale sequence comparison. Substring-based seeding methods such as kmers are ideal for sequences with low error rates but struggle to achieve high sensitivity while maintaining a reasonable precision for error-prone long reads. SubseqHash, a novel subsequence-based seeding method we recently developed, achieves superior accuracy to substring-based methods in seeding sequences with high mutation/error rates, while the only drawback is its computation speed.

**Results:**

We propose SubseqHash2, an improved algorithm that can compute multiple sets of seeds in one run, by defining *k* orders over all length-*k* subsequences and finding the optimal subsequence under each of the *k* orders in a single dynamic programming framework. The algorithm is further accelerated using single instruction, multiple data instructions for parallel computing. The design of SubseqHash2 also allows it to generate the same sets of seeds for a string and its reverse complement by using symmetric random tables. We demonstrate that SubseqHash2 drastically outperforms popular substring-based methods including kmers, minimizers, syncmers, and Strobemers for three fundamental applications. In read mapping, SubseqHash2 can generate adequate seed matches for aligning hard reads that minimap2 fails on. In sequence alignment, SubseqHash2 achieves high coverage of correct seeds and low coverage of incorrect seeds. In overlap detection, seeds produced by SubseqHash2 lead to more correct overlapping pairs at the same false-positive rate. In all experiments, SubseqHash2 achieves a 10–50× speedup over SubseqHash while maintaining nearly identical high accuracy. With all the algorithmic breakthroughs of SubseqHash2, we clear the path for the wide adoption of subsequence-based seeds in long-read analysis.

**Availability and implementation:**

SubseqHash2 is available at https://github.com/Shao-Group/SubseqHash2 and have also been archived on Software Heritage (swh:1:dir:86738fc4b919eb6a9a26f7f533c25eb69f9a96d5).

## 1 Introduction

The ever-growing, gigantic size of sequencing data poses a great challenge for sequence comparison. Seeding is a powerful technique that avoids the expensive computation of all-versus-all, full-length comparisons and is thus widely used in scalable methods. Broadly speaking, seeding transforms a long sequence into a list of shorter ones, often of a regular length, known as seeds. Seeds across sequences can be matched (i.e. compared for identity) in constant time with hash tables. A matching pair of seeds, commonly referred to as a seed match or anchor, indicates a potential biological relevance and suggests a candidate mapping location. The collected seed matches may be processed differently to fulfill specific tasks. For example, the seed-chain-extend strategy finds collinear chains of seed matches to maximize a predefined scoring function. This strategy, often combined with a fine-grained local alignment procedure to fill up the gaps between chained seed matches, has been widely adopted in read mapping and sequence alignment (Altschul *et al.* 1990, Myers and Miller 1995, Abouelhoda and Ohlebusch 2005, Jain *et al.* 2022) and is recently shown (Shaw and Yu 2022) theoretically to be both accurate and efficient. Another example is identifying all overlapping pairs in a large set of sequences, a critical and the most time-consuming step in constructing the overlap/string graph (Koren *et al.* 2017, Nurk *et al.* 2020, Cheng *et al.* 2021) for genome assembly (following the overlap-layout-consensus paradigm). For this task, seed matches are used to bucket sequences, thereby confining the search for overlapping pairs within individual buckets and significantly reducing the number of pairs that need to be compared. All these applications demand a seeding scheme that admits both high sensitivity, namely producing many seed matches on biologically related or similar sequences, and high precision, i.e. producing fewer or ideally zero seed matches on dissimilar sequences. Both properties are desirable in downstream analyses for producing accurate outcomes; high precision often also implies a reduction of running time.

Substring-based seeding methods, most notably kmers (i.e. substrings of length *k*), have been predominant in sequence analysis, mainly due to their simplicity and extraordinary performance on data with low error rates. However, when comparing sequences with high error rates such as homologous genes from distant species or PacBio and Oxford Nanopore long reads, the choice of *k* is usually full of compromises and frustrations. Using a large *k* guarantees high precision but is prohibitive for its extremely low sensitivity. Conversely, a small *k*, improves sensitivity, but because unrelated sequences may share many short kmers by chance, it often results in poor precision. This inherent dilemma of kmers makes it much less effective when the mutation/error rate is high. Many tools are forced to use a small *k* and compensate for the low precision by fine-grained follow-up steps such as chaining to filter out false positives, which significantly increases processing time and highlights the deficiency of using kmers for seeding. Existing sketching methods such as minimizers (Schleimer *et al.* 2003, Roberts *et al.* 2004, Marçais *et al.* 2018) and syncmers (Edgar 2021) can reduce the number of seeds and seed matches but cannot solve the sensitivity–precision perplexity of kmers (see the comparisons of kmers, minimizers, and syncmers in Figs 4–6). Spaced seeds (Califano and Rigoutsos 1993, Ma *et al.* 2002) and indel seeds (Mak *et al.* 2006) accommodate errors by masking positions with specific patterns, but only mutations at designated positions can be handled. Another strategy that recently gains popularity is to combine multiple short kmers to produce a longer seed; examples include neighboring minimizer pairs (Chin and Khalak 2019), Order Min Hash (Marçais *et al.* 2019), and Strobemers (Sahlin 2021, Maier and Sahlin 2022, Sahlin 2022). Nonetheless, as these methods are all substring-based, they cannot fully resolve the intrinsic weakness of kmers against mutations. Additionally, sketching and combining can be considered orthogonal to the basic seeding methods in the sense that they can be applied with any basic seeding scheme (see Section 2.5).

**Figure 1.**
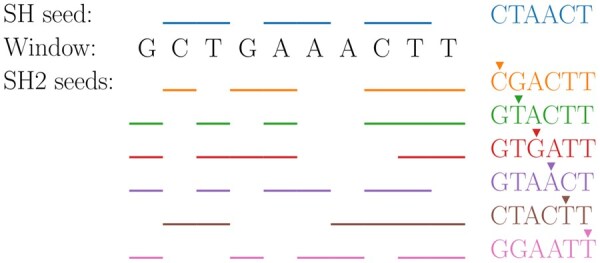
An illustration of SubseqHash and SubseqHash2 seeds from a window of length n=10 with seed length k=6. The bars label the chosen positions of the corresponding seed. For each SubseqHash2 seed, the pivot position is marked. The seeds are computed using the example tables in Tables 1–3, available as supplementary data at *Bioinformatics* online.

In Li *et al.* (2023), we proposed a novel subsequence-based seeding approach named SubseqHash. The key intuition is that two strings have a small edit distance *if and only if* they share long subsequences. More concretely, two length-*n* strings with an edit distance of *e* must share subsequence(s) of length at least n−e, and two length-*n* strings sharing a subsequence of length *k* must admit an edit distance at most 2(n−k). In contrast, two length-*n* strings with an edit distance of *e* can only guarantee to share a substring of length n−ke, as a single edit can break *k* continuous kmers. SubseqHash is defined as a function $h_{\pi }(x)$ that maps a length-*n* string x to its smallest length-*k* subsequence according to an order (i.e. permutation) π over all length-*k* strings, see Fig. 1 for an example. Formally, $h_{\pi }(x)=\arg\min_{z\in S_{k}(x)}\pi (z)$, where $S_{k}(x)$ denotes the set of length-*k* subsequences of x and we use π(z) to denote the ranking of z in the order π. Consider two length-*n* strings x and y. If π is drawn uniformly at random from all possible permutations of length-*k* strings, then according to the property of MinHash, the probability of hash collision equals to the Jaccard similarity of the two sets of subsequences, formally written as $\mathrm{Pr}(h_{\pi }(x)=h_{\pi }(y))=J_{k}(x,y)$, where $J_{k}(x,y):=|S_{k}(x)\cap S_{k}(y)|/|S_{k}(x)\cup S_{k}(y)|$. As mentioned above, when x and y are similar (i.e. edit distance is small), they must share (long) subsequences, i.e. $S_{k}(x)\cap S_{k}(y)\neq \emptyset$, resulting in a strictly positive probability of hash collision: $\mathrm{Pr}(h_{\pi }(x)=h_{\pi }(y))>0$. Meanwhile, when x and y are dissimilar, they are likely to not share any long subsequences, leading to $\mathrm{Pr}(h_{\pi }(x)=h_{\pi }(y))=0$. Both sides are desirable for effectively tolerating errors.

The main challenge is the computation of $h_{\pi }(x):=\arg\min_{z\in S_{k}(x)}\pi (z)$. Note that $S_{k}(x)$ is of exponential size; it is intractable to follow the MinHash scheme for kmers, namely, enumerating all subsequences of x and picking the smallest one (under π). We made an algorithmic breakthrough to overcome this by designing a specific order π termed the ABC order, along with an algorithm to calculate $h_{\pi }(x)$ in O(nkd) time, where *d* is a parameter that balances the probability of hash collision and the running time. Since now π is not fully random (i.e. it is not drawn uniformly at random from all possible permutations) the above property that $\mathrm{Pr}(h_{\pi }(x)=h_{\pi }(y))=J_{k}(x,y)$ does not hold strictly. However, the ABC order is designed to closely approximate a fully random permutation, and we used experimental results to demonstrate that the probability of hash collision $\mathrm{Pr}(h_{\pi }(x)=h_{\pi }(y))$ is close to the Jaccard similarity $J_{k}(x,y)$.

SubseqHash inherently tolerates errors/edits by using subsequence as seeds, making it particularly suitable for seeding sequences with high error rates. Experimental results showed that SubseqHash achieved better accuracy than substring-based methods on data with high error rates. However, there is still room for improvement. Specifically, a single run of SubseqHash cannot achieve both high sensitivity and precision. To control false positives, we typically choose a large *n* (e.g. n=30,40,50) and a *k* close to *n* (e.g. k=0.7n,0.8n,0.9n), as dissimilar strings are not expected to share subsequences of that long and hence a hash collision will not happen, resulting in the desired high precision. But with such *n* and *k*, the probability of hash collision $\mathrm{Pr}(h_{\pi }(x)=h_{\pi }(y))$ is not high enough for similar strings x and y, since the portion of shared length-*k* subsequences measured by $J_{k}(x,y)$ is small, leading to low sensitivity. To address this, we proposed a new seeding scheme by *repeating* SubseqHash multiple times (say *t* times), with independent ABC orders. Suppose that *p* is the probability of a hash collision when invoking SubseqHash once, the probability of having at least one hash collision among the *t* pairs of seeds can be boosted to $1-{\left(1-p\right)}^{t}$. Note that for similar strings, *p* is strictly positive (thanks to that similar strings share long subsequences), thus sensitivity is markedly enhanced. Conversely, dissimilar strings that are unlikely to share any long subsequence, will still have a zero or negligibly small probability of hash collision, implying minimal precision degradation. SubseqHash combined with repeating *can* yield both high sensitivity and precision, as we experimentally demonstrated in Section 3.1 and the SubseqHash paper (Li *et al.* 2023).

However, the above seeding scheme is computationally inefficient. In practice, sliding windows of length *n* are used on long reads and SubseqHash is repeatedly applied to each window. Let *N* be the length of a long read and containing N−n+1=O(N) length-*n* windows. The overall running time of seeding for a single long read is O(Nnkdt), which could be prohibitive for certain applications. This computational demand limits the widespread use of SubseqHash. We address this challenge in this paper by introducing SubseqHash2, which seeds for a length-*n* long read with *t* repeats in O(Nnk) time, a *dt*-fold speedup over SubseqHash. The acceleration is achieved by two algorithmic innovations. First, we design the ABCk orders, which defines *k* orders, and a new dynamic programming algorithm that finds the optimal subsequences for the *k* orders simultaneously. A single run of this algorithm generates *k* seeds, providing a *t*-fold speedup over SubseqHash for any t≤k. Second, recognizing the independence of the subproblems in the dynamic programming algorithm, we leverage (single instruction, multiple data (SIMD) parallelism that solves *d* subproblems in parallel with one set of instructions. This gains another *d*-fold speedup, for *d* up to 32. In practice, SubseqHash2 may not experience exactly a *dt*-fold speedup due to overhead, but the acceleration scales with *dt*. For example, with n=30 and a typical choice of k=25 and d=31, the observed speedup is about 50×.

In addition to the improved efficiency, the design of SubseqHash2 allows for symmetries in the score function, enable a string and its reverse complement to produce the same sets of seeds. This feature is highly desirable in sequence analysis, as it effectively halves both running time and storage requirements. We also show that SubseqHash2 can be extended to handle multiple windows and be combined with kmers. In the three applications including read mapping, pairwise alignment, and overlap detection, SubseqHash2 obtains much higher accuracy compared with substring-based seeding methods; it consistently mirrors the seed quality and accuracy of SubseqHash but with a substantial reduction in running time.

## 2 Materials and methods

The key idea of SubseqHash2 is the introduction of a pivot position that bifurcates the length-*k* subsequence into two disjoint subsequences (see Fig. 1 for an example). For each pivot, a score function can be defined by amalgamating the scores of the two subsequences and the pivot position, resulting in *k* score functions (i.e. *k* orders). Intriguingly, the optimal subsequences under the *k* orders can be computed together in a single dynamic programming framework, which takes 1/k running time compared to SubseqHash. The algorithm is further accelerated by SIMD, which solves *d* subproblems simultaneously, leading to a *d*-fold speedup. We also introduce the two variants of SubseqHash2: SubseqHash2r and SubseqHash2w.

### 2.1 The ABCk orders

We define the *k* orders over $\Sigma^{k}$, termed the ABCk orders. They are governed by an integer *d* and nine tables: forward tables $A_{F},B_{F},C_{F}$, reverse tables $A_{R},B_{R},C_{R}$, and pivot tables $A_{P},B_{P},C_{P}$. Tables $A_{F}$ and $A_{R}$ are of dimension k×d×|Σ|, i.e. $A_{F},A_{R}\in Z^{k\times d\times |\Sigma |}$. Table $A_{P}$ is of dimension k×|Σ|, i.e. $A_{P}\in Z^{k\times |\Sigma |}$. Tables $B_{F}$ and $B_{R}$ are of dimension k×d×|Σ|, and table $B_{P}$ is of dimension k×|Σ|; each element in them is a pair, namely, $B_{F}[i][j][\sigma ],B_{R}[i][j][\sigma ],B_{P}[i][\sigma ]\in \{(+1,+1),(+1,-1),(-1,+1),(-1,-1)\}$, 1≤i≤k, 0≤j≤d−1, and σ∈Σ. Tables $C_{F}$, $C_{R}$, and $C_{P}$ are of dimension k×|Σ|, where $C_{F}[i][\sigma ],C_{R}[i][\sigma ],C_{P}[i][\sigma ]\in \{0,1,\ldots ,d-1\}$, 1≤i≤k and σ∈Σ. These tables can be filled either by picking values from their specific ranges randomly, or in a symmetric way for a desired property (Section 2.4). An example of an ABCk order is included in Note 5, available as supplementary data at *Bioinformatics* online.

These nine tables, once filled, determine *k* orders over $\Sigma^{k}$, named $\pi_{1},\pi_{2},\ldots ,\pi_{k}$. All these orders utilize forward functions $\psi_{F}$ and $\omega_{F}$, as well as reverse functions $\psi_{R}$ and $\omega_{R}$, which we describe now. The three forward tables are used in the forward functions, while the three reverse tables are used in the reverse functions. Let $s\in \Sigma^{l}$ be a string of length *l*, where l≤k. Write $s=s_{1}s_{2}\cdots s_{l}$. Denote the first and the second element in the pair $B_{F}[i][j][\sigma ]$ by $B_{F}[i][j]{\left[\sigma \right]}_{1}$ and $B_{F}[i][j]{\left[\sigma \right]}_{2}$, respectively; the same applies for the tables $B_{R}$ and $B_{P}$. Then the functions are defined by the following recurrences:

$$\begin{array}{c} \psi_{F}(s_{1}\cdots s_{l})=(\psi_{F}(s_{1}\cdots s_{l-1})+C_{F}[l][s_{l}])\text{mod}\;d,\\ \psi_{R}(s_{1}\cdots s_{l})=(\psi_{R}(s_{1}\cdots s_{l-1})+C_{R}[l][s_{l}])\text{mod}\;d;\\ \omega_{F}(s_{1}\cdots s_{l})=\omega_{F}(s_{1}\cdots s_{l-1})\cdot B_{F}[l][\psi_{F}(s_{1}\cdots s_{l})]{\left[s_{l}\right]}_{1}\\ +A_{F}[l][\psi_{F}(s_{1}\cdots s_{l})][s_{l}]\cdot B_{F}[l][\psi_{F}(s_{1}\cdots s_{l})]{\left[s_{l}\right]}_{2},\\ \omega_{R}(s_{1}\cdots s_{l})=\omega_{R}(s_{1}\cdots s_{l-1})\cdot B_{R}[l][\psi_{R}(s_{1}\cdots s_{l})]{\left[s_{l}\right]}_{1}\\ +A_{R}[l][\psi_{R}(s_{1}\cdots s_{l})][s_{l}]\cdot B_{R}[l][\psi_{R}(s_{1}\cdots s_{l})]{\left[s_{l}\right]}_{2}. \end{array}$$

The initial values are set to $\psi_{F}(s)=\psi_{R}(s)=0$ and $\omega_{F}(s)=\omega_{R}(s)=0$ for empty string s.

We now define the *k* orders $\pi_{i}$, i=1,2,…,k, over $\Sigma^{k}$. Each order $\pi_{i}$ is essentially a score function that maps a length-*k* string to a pair. Let $z=z_{1}z_{2}\cdots z_{k}\in \Sigma^{k}$. Intuitively, $\pi_{i}$ picks $z_{i}$ as the pivot and combines the forward function and the reverse function using the pivot tables. Formally, we define $\pi_{i}(z):=(\psi_{i}(z),\omega_{i}(z))$, where

$$\begin{array}{cc} \psi_{i}(z):=\left(\psi_{R}(z_{i-1}z_{i-2}\cdots z_{1}\right)\\ +C_{P}[i][z_{i}]+\psi_{F}(z_{i+1}z_{i+2}\cdots z_{k}))\mathrm{mod}d & \\ \omega_{i}(z):=\omega_{R}(z_{i-1}z_{i-2}\cdots z_{1})\cdot B_{P}[i]{\left[z_{i}\right]}_{1}+A_{P}[i][z_{i}]\\ +\omega_{F}(z_{i+1}z_{i+2}\cdots z_{k})\cdot B_{P}[i]{\left[z_{i}\right]}_{2}. & \end{array}$$

For any two $z_{1},z_{2}\in \Sigma^{k}$, we define $\pi_{i}(z_{1})<\pi_{i}(z_{2})$, i.e. $z_{1}$ is ranked before $z_{2}$ in order $\pi_{i}$, if and only if $\psi_{i}(z_{1})<\psi_{i}(z_{2})$, or $\psi_{i}(z_{1})=\psi_{i}(z_{2})$ and $\omega_{i}(z_{1})>\omega_{i}(z_{2})$.

Similar to the ABC order introduced by Li *et al.* (2023), the techniques of using ±1 in $B_{F}$, $B_{R}$, and $B_{P}$ tables, the modular operation, and the pivot splitting all aim for assigning shuffled scores to two strings with small edit distance, making similar strings distant in the resulting order. This is critical for achieving a higher probability of hash collision. Note that the *k* orders in the ABCk orders are not independent as they use shared forward and reverse functions, but we experimentally show that they behave independently in practice, and SubseqHash2 achieves almost identical results with repeating SubseqHash using independent tables (see Section 3).

### 2.2 Algorithm for computing seeds

A common practice in handling (long) sequences is to generate seeds for each of its sliding window (i.e. length-*n* substring). Here, we design an algorithm that finds seeds for all windows in a given long sequence at one time, which runs Θ(k) times faster than processing each sliding window separately. Let X be a sequence of length *N*, N>n. We use X[w|b]. to denote the length-*b* substring of *X* starting from position *w*, i.e. $X[w|b]:=X_{w}X_{w+1}\cdots X_{w+b-1}$. We define

$$\Pi_{i}[w]:=\underset{z\in S_{k}(X[w|n])}{\min}\pi_{i}(z),$$

 

$$z_{i}^{*}[w]:=\underset{z\in S_{k}(X[w|n])}{\text{argmin}}\pi_{i}(z)$$

for every 1≤i≤k and 1≤w≤N−n+1. The set $\left\{z_{i}^{*}[w]|1\leq i\leq k\right\}$ gives the *k* optimal seeds for window X[w|n], corresponding to the *k* orders $\left\{\pi_{i}|1\leq i\leq k\right\}$, while $\left\{\Pi_{i}[w]|1\leq i\leq k\right\}$ gives the optimal scores.

Given X, we design an algorithm to calculate $\Pi_{i}[w]$ and $z_{i}^{*}[w]$. The algorithm consists of two steps, *iterating* and *optimizing*, following a typical dynamic programming scheme. In the iterating step, subproblems are defined and solved with recurrences. In the optimizing step, the smallest subsequences under each order for every window are calculated. Different windows may reuse the same subproblems, making this algorithm Θ(k) times faster than processing windows separately as SubseqHash did. For the interest of space, we leave the details of this algorithm in Note 1, available as supplementary data at *Bioinformatics* online.

### 2.3 SIMD parallelism

The total running time of the above algorithm is O(Nnkd), as the iterating and optimizing steps require either filling or tracking back of a dynamic programming table of dimension N×n×k×d. Here, we use SIMD instructions to speed up as they are widely supported by modern CPUs that can leverage wide registers to perform operations on multiple data elements in parallel. Many dynamic programming algorithms are not feasible to use SIMD to speed up as the subproblems usually depend on each other. In our case, the subproblems in the last dimension of the table can be made independent, and hence these subproblems can be solved together with SIMD instructions. We leave the details of SIMD parallelism in Note 3, available as supplementary data at *Bioinformatics* online.

### 2.4 SubseqHash2r: variant for reverse complement

In sequence analysis, it is a much desired property to *not* distinguish a sequence and its reverse complement. We can achieve this property by using symmetric tables in an ABCk order so that a length-*n* string and its reverse complement can be mapped into the same set of *k* seeds. We refer to this variant of SubseqHash2 as SubseqHash2r. Note that this symmetry is enabled by the multiple orders defined in the ABCk order, and hence cannot be directly transferred to SubseqHash.

Let $x\in \Sigma^{n}$ be a length-*n* string and let $\bar{x}$ be its reverse complement. We aim for having that *i*th seed of x to be equal to the (k−i+1)th seed of $\bar{x}$, for every 1≤i≤k, formally:

$$\begin{array}{c} \arg\underset{z\in S_{k}(x)}{\min}\pi_{i}(z)=\arg\underset{z\in S_{k}(\bar{x})}{\min}\pi_{k-i+1}(z)\\ =\arg\underset{z\in S_{k}(x)}{\min}\pi_{k-i+1}(\bar{z}). \end{array}$$

This condition is met if $\pi_{i}(z)=\pi_{k-i+1}(\bar{z})$, for every 1≤i≤k. We derive the requirements on the ABCk tables in order to satisfy them in Note 4, available as supplementary data at *Bioinformatics* online.

### 2.5 SubseqHash2w: variant concatenating a substring

The recently developed method Strobemer extracts a substring and some minimizers from the subsequent windows and concatenates them as a seed (Sahlin 2021, 2022, Maier and Sahlin 2022). We apply this idea to SubseqHash2, creating a new variant termed SubseqHash2w. In SubseqHash2w, a seed is formed by two parts: a substring and the smallest subsequence from the following window. We use an additional parameter $k_{0}$ to specify the length of the preceding substring. For an input x of length $k_{0}+n$, SubseqHash2w extracts the first substring of length $k_{0}$, and then compute *k* seeds of length *k* from the remaining string of length *n* using the above algorithm. The leading substring of length $k_{0}$ will be concatenated with each of the *k* seeds, resulting in *k* seeds of length $k_{0}+k$. We later compare SubseqHash2w with Strobemer.

### 2.6 Selection of parameters

SubseqHash2 has parameters *n*, *k*, *d*, and *t*. The combination of *n* and *k* balances the sensitivity and precision of the produced seeds. For a fixed *n*, a larger *k* provides lower sensitivity and fewer false positives. Different values of *n* may have different performance, while larger *n* also takes more running time. The sensitivity can be increased if *d* is increased; with SubseqHash2 we can use large *d* as its running time is independent of *d* for up to d=32. Parameter *t* specifies the number of seeds in a window (length-*n* string), which should be in the range of [1, *k*]. The selection of *t* can be application-dependent; in general, data with higher error rates might need a larger *t* to produce enough seed matches. The algorithm is implemented to allowing for specifying *t*, in which case the optimizing step can be sped up to just generate *t* seeds. SubseqHash2w has an additional parameter $k_{0}$, which also balances sensitivity and precision. In the experiments, we use $k_{0}=k/2$.

## 3 Results

We first present an analysis of the probability of hash collision as a function of edit distance for different seeding methods, illustrating their basic properties. Next, we benchmark the running time of SubseqHash and SubseqHash2. Last, we compare different seeding methods for three applications: read mapping, pairwise sequence alignment, and overlap detection.

### 3.1 Comparison of probability of hash collision

Most seeding methods can be interpreted as a (hashing) function that maps a string (typically a window in a long sequence in practice) into a seed. For examples, minimizer maps a length-*n* string to its smallest length-*k* substring, and SubseqHash/SubseqHash2 maps a length-*k* string to its smallest length-*k* subsequence. It is desirable for such a seeding method *h* to be both sensitive and precise (aka locality-sensitive), i.e. the probability of hash collision Pr(h(x)=h(y)) is high (resp. low) if the edit distance between two strings x and y, written as edit(x,y), is small (resp. large). When repeating *h* by *t* times, a length-*n* string x will be mapped into *t* seeds, written as $h_{i}(x)$, 1≤i≤t, and a seeding method is desired to achieve a high probability of having at least one hash collision $\mathrm{Pr}(\vee_{1\leq i\leq t}(h_{i}(x)=h_{i}(y)))$ when edit(x,y) is small, and a high probability of not having any hash collision $\mathrm{Pr}(\wedge_{1\leq i\leq t}(h_{i}(x)\neq h_{i}(y)))$ when edit(x,y) is large.

The probability of hash collisions can be estimated using simulations. We randomly generate pairs of strings of length *n* over alphabet {A, C, G, T}. A simulated pair will be put into category *i* if the edit distance between them is *i*, i=1,2,…,10. We simulate 100 000 pairs in each category. We apply minimizer, SubseqHash, and SubseqHash2 on the simulated pairs with repetition (t=10 or t=k) and without repetition (t=1). Minimizer is repeated by using different random seeds in its hash function in defining the order for kmers. SubseqHash is repeated by using random, independent tables used in the ABC order. SubseqHash2 generates *t* sets of seeds in a single run. The frequency of hash collisions (at least one hash collision in the case of repetitions) in each category will be used to estimate the probability.

**Figure 2.**
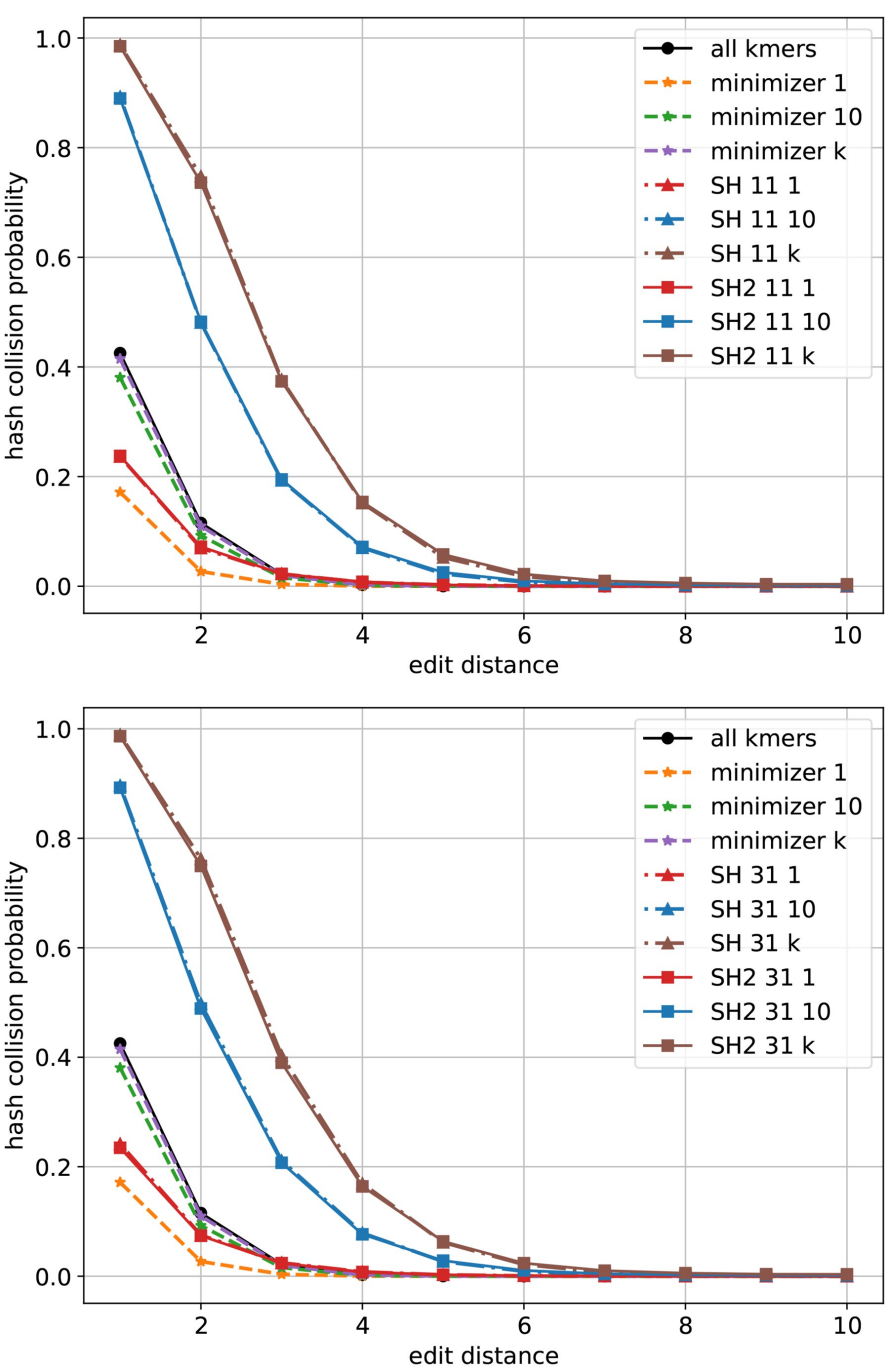
The probability of hash collision (at least one hash collision in the case of t=10 and t=k=24) estimated for different seeding methods with n=30 and k=24. “minimizer *t*” represents repeating minimizer *t* times. “SH *d t*” and “SH2 *d t*” represent SubseqHash and SubseqHash2 with parameter *d* and repeating *t* times. Results with different *n* and *k* are given in Figs 2–4, available as supplementary data at *Bioinformatics* online.

**Figure 3.**
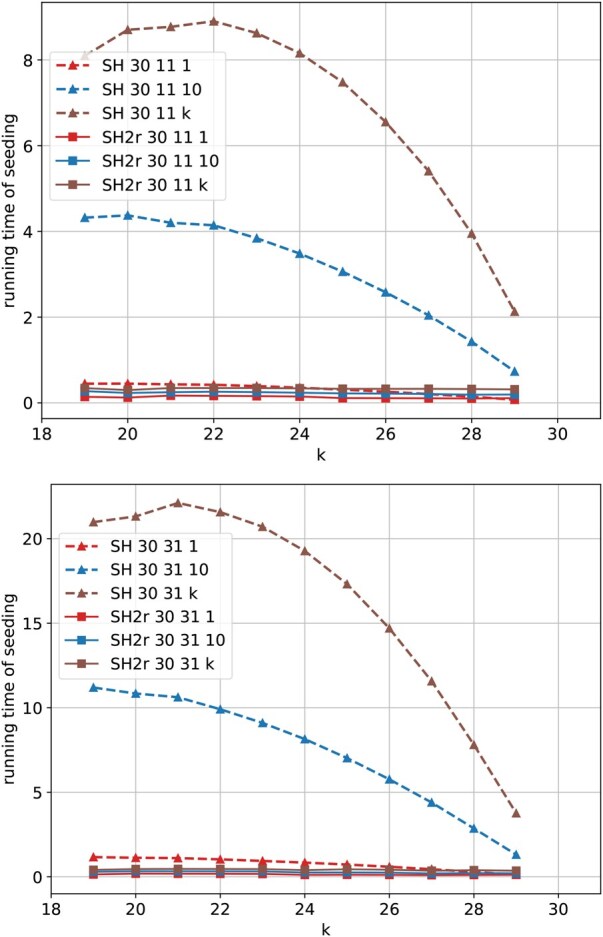
The average CPU time (second) of SubseqHash and SubseqHash2 per read. “SH *n d t*” and “SH2r *n d t*” represent SubseqHash and SubseqHash2r with window size of *n*, parameter *d*, and repeating *t* times; for both cases the lines connect points with varying *k*.

**Figure 4.**
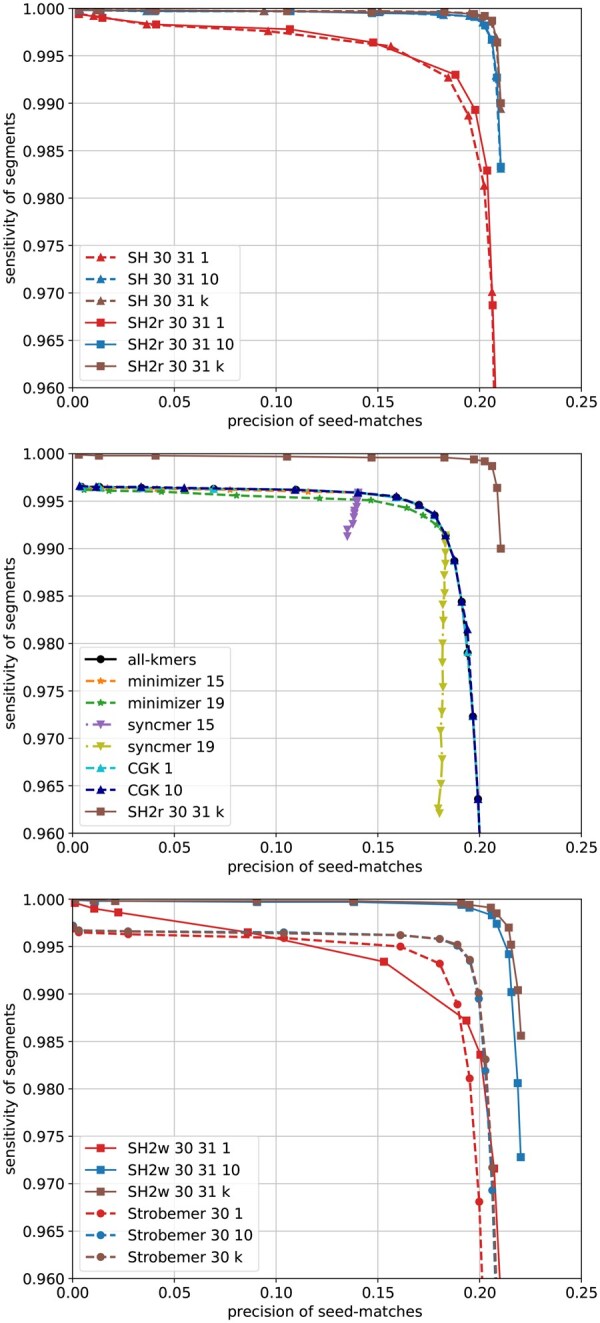
Comparison of average seed match precision and segment sensitivity using a simulated dataset modeled on the sequencing error profile of the Oxford Nanopore dataset SRX27559270. In the top figure, “SH *n d t*” and “SH2r *n d t*” represent SubseqHash and SubseqHash2r with window size of *n*, parameter *d*, and repeating *t* times; for both cases the lines connect points with varying *k* from 19 to 29. In the middle figure, the line “all-kmers” connects points with *k* from 10 to 25; “minimizer *n*” represents a line with window size of *n* and varying *k*. “syncmer *k*” represents closed syncmer with a length-*k* seed; the line connects points with varying *s* from 2 to k−1; “CGK *t*” represents using CGK-embedding for all kmers and repeating *t* times. In the bottom figure, “SH2w *n d t*” represents SubseqHash2w with window size of *n*, parameter *d*, and repeating *t* times; $k_{0}=k/2$. “Strobemer *n t*” represents Strobemer (two windows) with window size of *n* and repeating *t* times; the line connects points with varying parameter *k* from 5 to 20. Different methods may have different range of *n* and *k* to make their curves clear and comparable on the figure.

The estimated probabilities are shown in Fig. 2 and Figs 2–4, available as supplementary data at *Bioinformatics* online. First, observe that SubseqHash2 and SubseqHash with the same number of repeats exhibits nearly identical probability. This is exactly the goal of SubseqHash2: the same performance as SubseqHash but running in a much reduced time complexity. Second, repeating is effective for SubseqHash2 and SubseqHash, demonstrated by the facts that the probability can be drastically increased with small edit distance, while remaining nearly zero with large edit distance. On the contrary, repeating is not effective for minimizer, as the probability remains low even with small edit distance. We also included the results of using all kmers as seeds (therefore a pair of string has at least one hash collision with “all kmer” if they share at least one kmer). As expected, the probability of repeating minimizers is close to and bounded by that of using all kmers. For experiments in the following sections, we simply include the results of “all kmers” to approximate the results of repeating minimizer.

### 3.2 Comparison of running time

**Figure 5.**
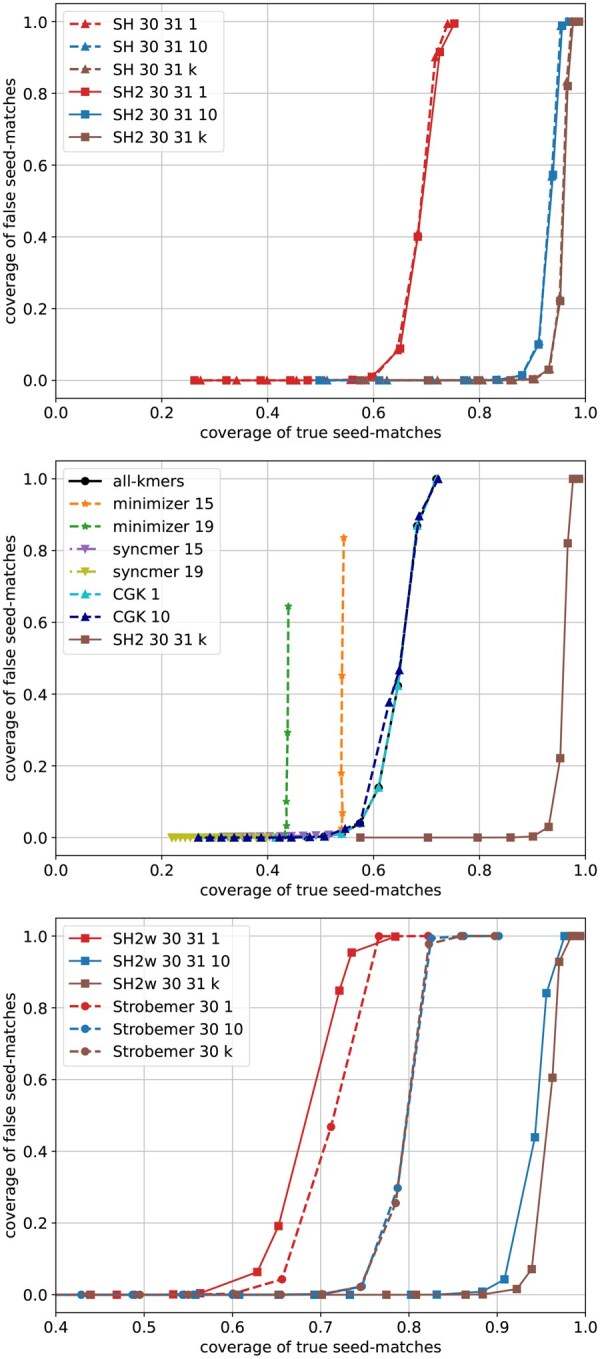
The true and false coverages of different seeding methods on data with error rate r=10%. The same parameters as in Section 3.3 are used.

We compare the running time of SubseqHash and SubseqHash2 in Fig. 3 and Fig. 5, available as supplementary data at *Bioinformatics* online. Both methods are applied to the SRX27559270 dataset (with Oxford Nanopore long reads), and we report the average CPU time per read. With the same parameters *d* and *t*, SubseqHash2 can be 50 times faster than SubseqHash. SubseqHash2 repeating *k* times can be even faster than SubseqHash without repeating. It is also noteworthy that the running time of SubseqHash2 only experiences a slight increase as *d* and *t* grow, suggesting that the efficiency of SubseqHash2 remains nearly independent of *d* and *t*, confirming our theoretical analysis that the time complexity is substantially improved.

We also compare with kmer-based methods on the same dataset. Not surprisingly, kmers, Minimizers, and syncmers are exceedingly fast, taking less than 5 s across almost all choices of parameters, as they only need a linear scan of each read. Strobemers at a typical configuration (two strobes of length 15 within a window of size 50) consumes 145.19 s with 10 repeats. In comparison, SubseqHash2, configured with n=30, k=25, d=31, and 10 repeats, uses 577.45 s, placing it in a comparable time range as Strobemers. Notably, SubseqHash, with the identical (n,k,d,t) as SubseqHash2, requires a substantially longer running time of 15 364 s to complete the task. We note that repeat is a sensible option for both Strobemers and SubseqHash2, in order to achieve their best performances in three following applications.

In terms of memory usage, all methods except SubseqHash2 require less than 10 MB during the seeding stage on this dataset. While SubseqHash2 incurs additional memory allocated for dynamic programming tables, the total memory usage of SubseqHash2 remains under 500 MB when performing seeding on long reads, which can be easily accommodated on modern computers. The detailed memory usage can be found in Table 4, available as supplementary data at *Bioinformatics* online.

### 3.3 Read mapping

We now consider the task of mapping error-prone long reads to reference genomes. For this purpose, we simulate a dataset using PBSIM (Ono *et al.* 2022) from chromosome X of *Drosophila melanogaster* (NC_004354.4). The simulation is performed with the sampling-based mode, where a recent Oxford Nanopore R10 dataset SRX27559270 is provided to the simulator to mimic the statistical characteristics of real reads. [Results on another simulation with a higher error rate according to the error profile of a PacBio dataset SRX499318 used by Song *et al.* (2020) are shown in Figs 7 and 8, available as supplementary data at *Bioinformatics* online.] Reads that share long substrings with the parts of the reference where they originate can be mapped easily using either seeding methods. The real challenge lies in identifying true seed matches for reads (or parts of reads) that barely share any meaningfully long substring with the reference. Our seeding method is designed to tackle such “hard” reads. We argue that this is a valid and intended pipeline—it would be unwise to completely abandon the agile substring-based methods with existing highly optimized implementations such as minimap2 (Li 2018) for the easier tasks; instead, we use them as a filter to expose cases where only subsequence-based seeds can provide a satisfactory solution. From all the simulated reads, around 5000 unmapped read fragments for each dataset from the results of minimap2 that are longer than 1000 bp are kept for our analysis.

SubseqHash2 is compared with minimizer, closed syncmer, CGK-embedding (Chakraborty *et al.* 2016), and all-kmers, for generating seeds on both the reads and the reference chromosome. As we do not know the strand of reads, we use SubseqHash2r, with one run being able to cover both strands; other methods need to be applied both on the reads and their reverse complements. All seeding methods are configured to generate seeds of length *k*, with the sole exception of CGK-embedding, which is applied to all kmers to produce a 3*k*-dimensional embedding for each kmer; seed matches are then collected between the reads and the reference. When repeats are used, seed matches from repeated runs are pooled. Note that a seed match specifies *k* aligned characters across the two sequences. We define a seed match to be *true* if over 50% of its *k* aligned characters coincide with the ground truth which we know through simulation; otherwise, we call it a false seed match.

In the seed-extend or seed-chain-extend framework of read mapping, more true seed matches and fewer false seed matches are definitely desirable. We therefore report the *precision of seed matches*, defined as the number of true seed matches divided by the total number of seed matches. Additionally, it is also crucial that these seed matches can be evenly distributed across the read, ensuring sufficient information for chaining and producing a complete alignment. To assess this, we partition all reads into segments of length 200 bp. An ideal seeding would cover every segment with true seed matches. We hence also report a *segment sensitivity*, defined as the percentage of segments with at least one true seed match.

**Figure 6.**
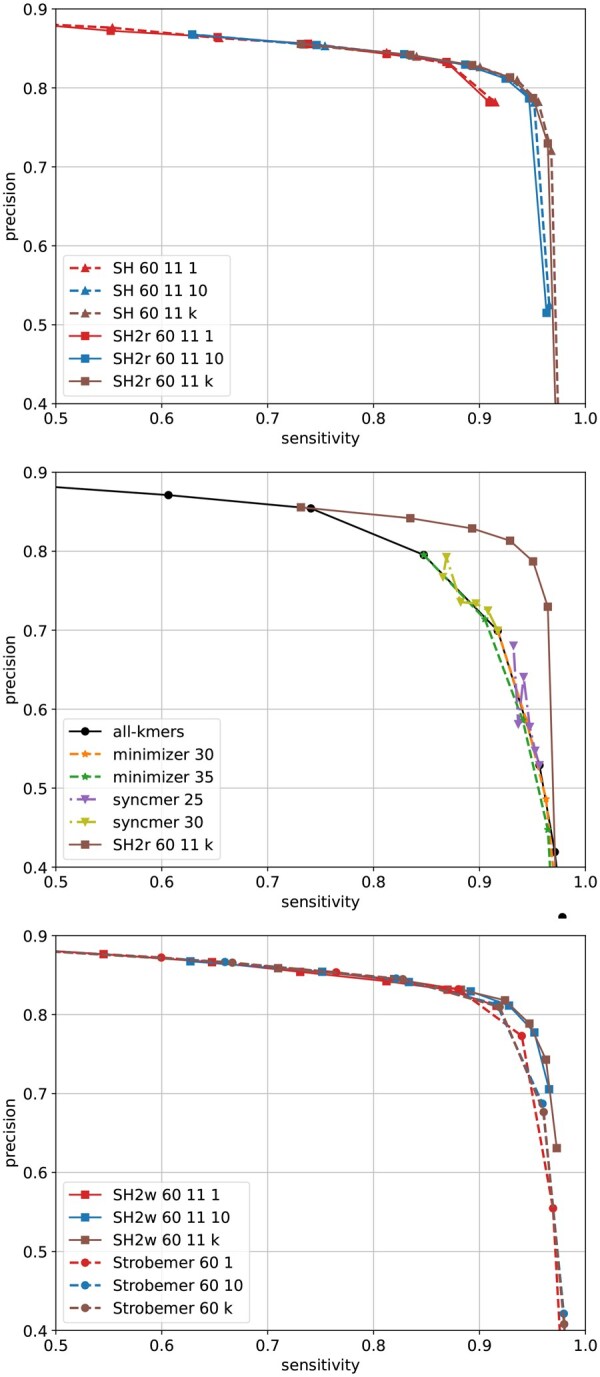
Overlap detection results on 10 000 reads sampled from the *E. coli* SRX27559270 dataset with window size 60 and d=11. The top figure shows that SubseqHash2 produces equally high-quality seeds as SubseqHash. The middle and bottom figures demonstrate that SubseqHash2 seeds enable more accurate overlap detection than other methods. The figures are cropped to only show the high sensitivity and precision regions.

In Fig. 4 and Figs 6–8, available as supplementary data at *Bioinformatics* online, we present these metrics acquired by various methods. Observe that the segment sensitivity of substring-based methods drops rapidly as *k* increases. This is because in these “hard” reads the exact match of long kmers with reference is infrequent, as otherwise minimap2 would align them correctly. In contrast, SubseqHash2 with repeats demonstrates the ability to capture adequate true seed matches while precision increases, resulting in a curve significantly above others. Furthermore, the curves of substring-based methods confirm that on “hard” regions, no choice of *k* can produce a satisfactory balance between sensitivity and precision, whereas SubseqHash2 with repeats maintains high sensitivity across the precision spectrum. This attests to the superiority of SubseqHash2 in generating high-quality seeds for aligning difficult reads.

We compare SubseqHash2w with Strobemer (Sahlin 2021, Randstrobe with two windows), as both employ multiple windows. To repeat Strobemer, we change the random seed in its hash function. Without repeating SubseqHash2w shows slightly better performance. However, with repetitions, SubseqHash2w demonstrates a substantial improvement, highlighting the effectiveness of repetition in SubseqHash and SubseqHash2.

### 3.4 Pairwise sequence alignment

We then evaluate the performance of seeding methods for pairwise sequence alignment through simulations. For each error rate r=0.05,0.1,0.15, we simulate 10 pairs of long sequences and report the average measures. We simulate a pair by randomly generating a sequence of length L=1 000 000 as its first sequence, followed by applying an edit, with probability of r/3 being a substitution, insertion, or deletion, at each position, to get its second sequence. We compute the *coverage* of true (resp. false) seed matches, defined as the proportion of characters encompassed by true (resp. false) seed matches. High true coverage and low false coverage are desirable, as the former implies long range of correct alignment, while the latter implies low chance of producing incorrect alignment. These two measures can be interpreted as sensitivity and precision of a seeding method for sequence alignment.

We compare SubseqHash2 with other four methods: minimizers, closed syncmer, all-kmers, and SubseqHash. The results are given in Fig. 5 and Figs 9–13, available as supplementary data at *Bioinformatics* online. Again, SubseqHash2 mirrors the accuracy from SubseqHash at the same level of repetitions, as we aimed for. With repetitions, SubseqHash2/SubseqHash considerably outperforms substring-based methods at all error rates. The results of SubseqHash2w and Strobemer are also shown. Although Strobemer performs better without repeating, SubseqHash2w significantly outperforms with repetitions.

### 3.5 Overlap detection

Overlap graph is a fundamental data structure used by many long-read genome assemblers where each pair of overlapping reads are connected. Accurately identifying such pairs is thus crucial: having too many false positives tends to produce a tangled graph, while missing true positives causes gaps in the assembly. Seeding methods are used to alleviate the computational burden by efficiently filtering out most of the nonoverlapping pairs: each read is transformed into a set of seeds, and pairs of reads are considered candidates for further verification only if they share a common seed. Usually, heuristics are applied such as filtering out high-frequency seeds, requiring more seed matches to trigger a candidate pair, chaining colinear seed matches, and applying other fine-grained verification methods (e.g. local alignment), all of which can improve the final results. We note that such preprocessing and postprocessing steps are independent of the choice of seeds, so we omit them in this experiment to have a direct comparison of performances among different seeding schemes.

A recent Oxford Nanopore long read dataset SRX27559270 (*Escherichia coli*) is used. Reads are first mapped to the reference genome with minimap2 to construct ground truth for evaluation. Reads with a unique high-quality mapping (quality score 60) are kept, from which 10 000 reads are sampled. The reads are then trimmed to only keep the mapped portion. A pair of reads are considered truly overlap (i.e. ground truth) if their mapped regions on the reference overlap by at least 15 bp. Sensitivity and precision are defined as the fractions of reported pairs that are correct over all ground truth pairs and over all reported pairs, respectively. In order to identify overlapping reads from opposite strands, with the exception of SubseqHash2r, other methods collect seeds twice from each read, one for each strand.

The precision–sensitivity curves are compared in Fig. 6. Detailed parameters for each compared method are recorded in Note 6, available as supplementary data at *Bioinformatics* online. It is not surprising that all methods can achieve near-perfect sensitivity by shortening the seed length, but this comes at the cost of excessive false positives, which defeats the purpose of seeding. Similarly, all methods can reach high precision with longer seeds, but this results in many true overlapping pairs being missed. SubseqHash/SubseqHash2 provide significantly higher sensitivity than substring-based seeds at comparable precision levels, particularly in the high-precision range. For substring-based seeds, the extreme of repeating is to include all-kmers, which produces little improvement. Repeating helps (random) Strobemers but only to a limited extent (observe that Strobemer curves with 10 and *k* repeats almost overlap). This is because its components are kmers and hence have a restricted range of choices; furthermore, all the kmers in two similar windows can be easily destroyed by just a few errors, in which case no matching Strobemers can be found regardless of the number of repeats. In contrast, the performance of SubseqHash/SubseqHash2 can still benefit from more repeats, justifying the motivation of producing *k* seeds in one run.

Notably, SubseqHash/SubseqHash2 yields even greater gains on more difficult datasets with higher error rates. Results on such more challenging datasets are shown in Figs 14 and 15, available as supplementary data at *Bioinformatics* online.

## 4 Conclusion

We introduced SubseqHash2, an advanced subsequence-based seeding method. Remarkably, it can be 50 times faster than its predecessor, SubseqHash, while preserving high seed quality. The innovative strategy of using a pivot position allows SubseqHash2 to produce up to *k* seeds for a window in a single pass. This approach substantially reduces the computational time of producing t≤k sets of seeds from O(Nnkdt) to O(Nnkd) and grants SubseqHash2 the flexibility to adapt its seed count to enhance performance across various sequence analysis tasks without incurring additional computation cost. Additionally, we employ SIMD instructions in the dynamic programming algorithm for seed computation, enabling parallel computation of *d* subproblems and further reducing the dynamic programming running time to O(Nnk). Throughout our experiments, SubseqHash2 matches the performance of SubseqHash while delivering a significant reduction in actual running time. Moreover, these algorithmic innovations enable generating the same sets of seeds for a sequence and its reverse complement, which further halves the running time for reads with unknown strand information. SubseqHash2 is versatile, can be combined with kmers in seeding as needed.

## 5 Discussion

We recognize that the superior seed quality of our approach comes at the cost of a more complicated algorithm, and therefore, despite being substantially improved, is still slower comparing to the simple and fast substring-based seeding methods. We are actively exploring ideas for further acceleration, both in terms of algorithmic design and implementation optimization. On the other hand, we believe strategically combining the agility of substring-based methods and the high sensitivity of subsequence-based seeding can potentially produce an ideal solution for analyzing data with high mutation/error rates, as demonstrated by our read mapping experiment. Given the unsatisfied accuracy of substring-based methods on error-prone data, as well as the common struggle of choosing *k*, we are optimistic that SubseqHash2, as a less swift but more accurate alternative, can establish its value across a broader spectrum of applications.

## Supplementary Material

btaf418_Supplementary_Data

## Data Availability

The real data used in this article are available through the Sequence Read Archive (SRA) at https://www.ncbi.nlm.nih.gov/sra and can be accessed using the accession numbers SRX27559270 and SRX27559270, which are also referenced in the respective sections.

## References

[btaf418-B1] Abouelhoda MI , Ohlebusch E. Chaining algorithms for multiple genome comparison. J Discrete Algorithms 2005;3:321–41.

[btaf418-B2] Altschul SF , Gish W, Miller W et al Basic local alignment search tool. J Mol Biol 1990;215:403–10. 2231712 10.1016/S0022-2836(05)80360-2

[btaf418-B3] Califano A , Rigoutsos I. FLASH: a fast look-up algorithm for string homology. In: *Proceedings of IEEE Conference on Computer Vision and Pattern Recognition (CVPR'93)*, *New York, NY, USA*. IEEE, 1993, 353-9. 10.1109/CVPR.1993.341106 7584371

[btaf418-B4] Chakraborty D , Goldenberg E, Kouck M. Streaming algorithms for embedding and computing edit distance in the low distance regime. In: *Proceedings of the 48th ACM Symposium on Theory of Computing (STOC’16), Cambridge, MA, USA*. New York, NY, USA: ACM, 2016, 712–25. 10.1145/2897518.2897577

[btaf418-B5] Cheng H , Concepcion GT, Feng X et al Haplotype-resolved de novo assembly using phased assembly graphs with hifiasm. Nat Methods 2021;18:170–5. 33526886 10.1038/s41592-020-01056-5 PMC7961889

[btaf418-B6] Chin C-S , Khalak A. Human genome assembly in 100 minutes. *bioRxiv*, 2019, preprint: not peer reviewed.

[btaf418-B7] Edgar R. Syncmers are more sensitive than minimizers for selecting conserved *k*-mers in biological sequences. PeerJ 2021;9:e10805. 33604186 10.7717/peerj.10805 PMC7869670

[btaf418-B8] Jain C , Gibney D, Thankachan SV. Co-linear chaining with overlaps and gap costs. In: *Proceedings of the 26th International Conference on Computional Molecular Biology (RECOMB’22), San Diego, CA, USA*. Switzerland: Springer, Cham, 2022, 246–62.

[btaf418-B9] Koren S , Walenz BP, Berlin K et al Canu: scalable and accurate long-read assembly via adaptive *k*-mer weighting and repeat separation. Genome Res 2017;27:722–36. 28298431 10.1101/gr.215087.116 PMC5411767

[btaf418-B10] Li H. Minimap2: pairwise alignment for nucleotide sequences. Bioinformatics 2018;34:3094–100. 29750242 10.1093/bioinformatics/bty191 PMC6137996

[btaf418-B11] Li X , Shi Q, Chen K et al Seeding with minimized subsequence. Bioinformatics 2023;39:i232–241. 37387132 10.1093/bioinformatics/btad218 PMC10311335

[btaf418-B12] Ma B , Tromp J, Li M. Patternhunter: faster and more sensitive homology search. Bioinformatics 2002;18:440–5. 11934743 10.1093/bioinformatics/18.3.440

[btaf418-B13] Maier BD , Sahlin K. Entropy predicts sensitivity of pseudorandom seeds. *Genome Res* 2023;33(7):1162–74. 10.1101/gr.277645.123 PMC10538493 37217253

[btaf418-B14] Mak D , Gelfand Y, Benson G. Indel seeds for homology search. Bioinformatics 2006;22:e341–e349. 16873491 10.1093/bioinformatics/btl263

[btaf418-B15] Marçais G , DeBlasio D, Kingsford C. Asymptotically optimal minimizers schemes. Bioinformatics 2018;34:i13–22. 29949995 10.1093/bioinformatics/bty258 PMC6037127

[btaf418-B16] Marçais G , DeBlasio D, Pandey P et al Locality-sensitive hashing for the edit distance. Bioinformatics 2019;35:i127–35. 31510667 10.1093/bioinformatics/btz354 PMC6612865

[btaf418-B17] Myers G , Miller W. Chaining multiple-alignment fragments in sub-quadratic time. In: *Proceedings of the 6th ACM-SIAM Symposium on Discrete Algorithms (SODA’95), San Francisco, CA, USA*, Philadelphia, PA, USA: SIAM, 1995, 38–47.

[btaf418-B18] Nurk S , Walenz BP, Rhie A et al HiCanu: accurate assembly of segmental duplications, satellites, and allelic variants from high-fidelity long reads. Genome Res 2020;30:1291–305. 32801147 10.1101/gr.263566.120 PMC7545148

[btaf418-B19] Ono Y , Hamada M, Asai K. Pbsim3: a simulator for all types of pacbio and ont long reads. NAR Genom Bioinform 2022;4:lqac092. 10.1093/nargab/lqac092 36465498 PMC9713900

[btaf418-B20] Roberts M , Hayes W, Hunt BR et al Reducing storage requirements for biological sequence comparison. Bioinformatics 2004;20:3363–9. 15256412 10.1093/bioinformatics/bth408

[btaf418-B21] Sahlin K. Effective sequence similarity detection with Strobemers. Genome Res 2021;31:2080–94. 34667119 10.1101/gr.275648.121 PMC8559714

[btaf418-B22] Sahlin K. Strobealign: flexible seed size enables ultra-fast and accurate read alignment. Genome Biol 2022;23:260–27. 36522758 10.1186/s13059-022-02831-7 PMC9753264

[btaf418-B23] Schleimer S , Wilkerson DS, Aiken A. Winnowing: local algorithms for document fingerprinting. In: *Proceedings of the 2003 ACM SIGMOD International Conference on Management of Data (SIGMOD/PODS’03), San Diego, CA, USA*. New York, NY, USA: ACM, 2003, 76–85. 10.1145/872757.872770

[btaf418-B24] Shaw J , Yu YW. Proving sequence aligners can guarantee accuracy in almost *O*(*m* log *n*) time through an average-case analysis of the seed-chain-extend heuristic. *Genome Res* 2023;33(7):1175–87. 10.1101/gr.277637.122 PMC10538486 36990779

[btaf418-B25] Song Y , Tang H, Zhang H et al Overlap detection on long, error-prone sequencing reads via smooth q-gram. Bioinformatics 2020;36:4838–45. 32311007 10.1093/bioinformatics/btaa252

