# Supplementary material for: Efficient seeding for error-prone sequences with SubseqHash2
Source: Bioinformatics. 2025 Jul 24;41(8):btaf418. doi: 10.1093/bioinformatics/btaf418 (PMC12317743; doi:10.1093/bioinformatics/btaf418)
Supplement: btaf418_Supplementary_Data [file btaf418_supplementary_data.pdf]

Supplementary Materials for
“Efficient Seeding for Error-Prone Sequences with SubseqHash2”

Xiang Li[1], Ke Chen[1], and Mingfu Shao[1,2,*]

[1]Department of Computer Science and Engineering, School of Electronic Engineering and Computer Science, The Pennsylvania State University

[2]Huck Institutes of the Life Sciences, The Pennsylvania State University

July 20, 2025

List of Supplementary Notes

1 Algorithm for calculating seeds 2
2 Algorithm for calculating $\Psi_i[w][b]$ 5
3 SIMD parallelism 7
4 ABCk tables used by SubseqHash2r 8
5 An example for the ABCk order 9
6 Parameters for overlap detection 14

List of Supplementary Figures

1 Illustration of the algorithm for computing the seeds 2
2 Probability of hash collision for $n = 20$ and $k = 12, 14$. 15
3 Probability of hash collision for $n = 20$ and $k = 16, 18$. 16
4 Probability of hash collision for $n = 30$ 17
5 The average CPU time in read mapping. 18
6 Precision and sensitivity in read mapping on a simulated Oxford Nanopore dataset with $n = 40$. 19
7 Precision and sensitivity in read mapping on a simulated PacBio dataset with $n = 30$. 20
8 Precision and sensitivity in read mapping on a simulated PacBio dataset with $n = 40$. 21
9 True/false coverage in sequence alignment with error rate r= 5%, $n = 30$. 22
10 True/false coverage in sequence alignment with error rate r= 5%, $n = 40$. 23
11 True/false coverage in sequence alignment with error rate r= 10%, $n = 40$. 24
12 True/false coverage in sequence alignment with error rate r= 15%, $n = 30$. 25
13 True/false coverage in sequence alignment with error rate r= 15%, $n = 40$. 26
14 Overlap detection results on a Pacbio *E. coli* dataset with $n = 60$ 27
15 Overlap detection results on a Pacbio *D. melanogaster* dataset with $n = 60$ 28

List of Supplementary Tables

1 An example of random tables A_F, B_F, C_F used in an ABCk order 11
2 An example of random tables A_R, B_R, C_R used in an ABCk order 12
3 An example of random tables A_P, B_P, C_P used in an ABCk order 13
4 Peak memory usage in seed generation 29

*Correspondence should be addressed to mxs2589@psu.edu.

Supplementary Note 1: Algorithm for calculating seeds

Given a length-N sequence $\boldsymbol{X}$, we now describe the algorithm to calculate $\Pi_i[w]$ and $\boldsymbol{z}_i^*[w]$, i.e., the optimal scores and the optimal seeds, for every $1 \le i \le k$ and $1 \le w \le N - n + 1$.

For any string $\boldsymbol{x}$ we use $S_l(\boldsymbol{x})$ to denote the set of length-l subsequences of x, $l \le |\boldsymbol{x}|$. For each $1 \le w \le N - n + 1$, $1 \le b \le n$, $1 \le l \le k$, and $0 \le j < d$, we define subproblem $F_{\min}[w][b][l][j]$, where w and b specify window $X[w|b]$, l indicates that length-l subsequences of $X[w|b]$ are considered, j restricts that the ψ value of the subsequences being considered must be equal to j, and $F_{\min}[w][b][l][j]$ is defined as the smallest ω value among all such subsequences. Similarly we can also define subproblems $F_{\max}[w][b][l][j]$. Their formal definitions are given below.

$$\begin{aligned} F_{\min}[w][b][l][j] &:= \min\nolimits_{\boldsymbol{s} \in S_l(X[w|b]) \text{ and } \psi_F(\boldsymbol{s})=j} \, \omega_F(\boldsymbol{s}), \\ F_{\max}[w][b][l][j] &:= \max\nolimits_{\boldsymbol{s} \in S_l(X[w|b]) \text{ and } \psi_F(\boldsymbol{s})=j} \, \omega_F(\boldsymbol{s}). \end{aligned}$$

We also define subproblems $R_{\min}[w][b][l][j]$ and $R_{\max}[w][b][l][j]$, which are to compute the minimized and maximized ω value among all *reversed* length-l subsequences in window $X[w|b]$ given their ψ value must be equal to j. Their definitions are given below, in which we use $\overleftarrow{\boldsymbol{s}}$ to represent the reversed string of s (i.e., if $\boldsymbol{s} = ACGAT$ then $\overleftarrow{\boldsymbol{s}} = TAGCA$).

$$\begin{aligned} R_{\min}[w][b][l][j] &:= \min\nolimits_{\boldsymbol{s} \in S_l(X[w|b]) \text{ and } \psi_R(\overleftarrow{\boldsymbol{s}})=j} \, \omega_R(\overleftarrow{\boldsymbol{s}}), \\ R_{\max}[w][b][l][j] &:= \max\nolimits_{\boldsymbol{s} \in S_l(X[w|b]) \text{ and } \psi_R(\overleftarrow{\boldsymbol{s}})=j} \, \omega_R(\overleftarrow{\boldsymbol{s}}). \end{aligned}$$

The iterating-steps solves all above subproblems. Since both ψ and ω functions are recursively defined, it is straightforward to give the recurrences. Specifically, for $F_{\min}$ and $F_{\max}$ we consider if the last (the l-th) letter of the optimal $\boldsymbol{s}$ comes from the the last (i.e. X_{w+b-1}) letter of the window. Whether $F_{\min}$ or $F_{\max}$ gets used for the smaller subproblem depends on the binary vector in the corresponding B_F table. The

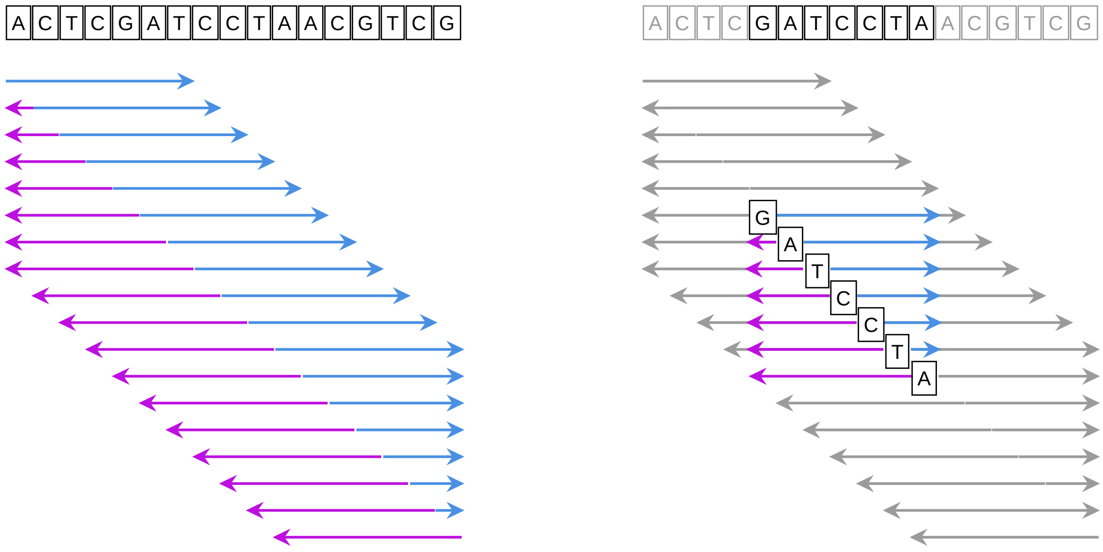

Supplementary Figure 1: Illustration of the algorithm computing seeds for each window of length $n = 7$ of the given long sequence (on top; length $N = 17$). The left panel shows the *iterating* step, in which the blue arrows show the $F_{\min}$ and $F_{\max}$ subproblems and purple arrows show the $R_{\min}$ and $R_{\max}$ subproblems. The right panel shows the *optimizing* step for a particular window (highlighted); the subproblems required for finding the optimal seeds in this window, which are available in the iterating step, are also highlighted.

recurrences are given below, in which we define $\sigma := X_{w+b-1}$ and $j' := (j - C_F[l][\sigma] + d) \bmod d$:

$$F_{\min}[w][b][l][j] = \min \begin{cases} F_{\min}[w][b-1][l][j] \\ A_F[l][j][\sigma] \cdot B_F[l][j][\sigma]_2 + \begin{cases} +F_{\min}[w][b-1][l-1][j'], & \text{if } B_F[l][j][\sigma]_1 = +1 \\ -F_{\max}[w][b-1][l-1][j'], & \text{if } B_F[l][j][\sigma]_1 = -1 \end{cases} \end{cases}$$

$$F_{\max}[w][b][l][j] = \max \begin{cases} F_{\max}[w][b-1][l][j] \\ A_F[l][j][\sigma] \cdot B_F[l][j][\sigma]_2 + \begin{cases} +F_{\max}[w][b-1][l-1][j'], & \text{if } B_F[l][j][\sigma]_1 = +1 \\ -F_{\min}[w][b-1][l-1][j'], & \text{if } B_F[l][j][\sigma]_1 = -1 \end{cases} \end{cases}$$

For $R_{\min}$ and $R_{\max}$ we consider whether the last letter of the optimal $\overleftarrow{\boldsymbol{s}}$, which is the first letter of $\boldsymbol{s}$, comes from the first letter of the window. Define $\sigma := X_w$ and $j' := (j - C_R[l][\sigma] + d) \bmod d$. We have

$$R_{\min}[w][b][l][j] = \min \begin{cases} R_{\min}[w+1][b-1][l][j] \\ A_R[l][j][\sigma] \cdot B_R[l][j][\sigma]_2 + \begin{cases} +R_{\min}[w+1][b-1][l-1][j'], & \text{if } B_R[l][j][\sigma]_1 = +1 \\ -R_{\max}[w+1][b-1][l-1][j'], & \text{if } B_R[l][j][\sigma]_1 = -1 \end{cases} \end{cases}$$

$$R_{\max}[w][b][l][j] = \max \begin{cases} R_{\max}[w+1][b-1][l][j] \\ A_R[l][j][\sigma] \cdot B_R[l][j][\sigma]_2 + \begin{cases} +R_{\max}[w+1][b-1][l-1][j'], & \text{if } B_R[l][j][\sigma]_1 = +1 \\ -R_{\min}[w+1][b-1][l-1][j'], & \text{if } B_R[l][j][\sigma]_1 = -1 \end{cases} \end{cases}$$

Initially, for any $1 \le w \le N-n+1$ and $1 \le b \le n$, $F_{min}[w][b][0][0] = F_{max}[w][b][0][0] = R_{min}[w][b][0][0] = R_{max}[w][b][0][0] = 0$ and $F_{min}[w][b][0][j] = F_{max}[w][b][0][j] = R_{min}[w][b][0][j] = R_{max}[w][b][0][j] = \text{NaN}$ if $j \neq 0$. When applying above recurrences to solve all subproblems, if any of the arithmetic operations $\{+, -\}$ involves NaN as an operand, then the result is also an NaN. The min and max operations ignore NaN and only work on numerical operands, unless there is none, in which case an NaN is returned. We essentially use NaN to indicate that no feasible subsequence exists for a subproblem. There are $O(Nnkd)$ subproblems defined; solving all of them using above recurrences takes $O(Nnkd)$ time.

The optimizing-step calculates the optimal score $\Pi_i[w] := \min_{\boldsymbol{z} \in S_k(X[w|n])} \pi_i(\boldsymbol{z})$ and the optimal subsequence $\boldsymbol{z}_i^*[w]$ under each order π_i for each window $X[w|n]$ by using the solutions of above subproblems. We enumerate where the i-letter of $\boldsymbol{z}_i^*[w]$, which is the pivot position for order π_i, comes from in $X[w|n]$. Having, say X_{w+b-1} is the i-the letter for a particular b, $1 \le b \le n$, then calculating $\Pi_i[w]$ becomes two smaller problems involving finding a length-$(i-1)$ subsequence $\boldsymbol{s_1}$ from $X[w|b-1]$ and a length-$(k-i)$ subsequence $\boldsymbol{s_2}$ from $X[w+b|n-b]$. Formally, we write

$$\Pi_i[w] = \min\nolimits_{1 \le b \le n} \Pi_i[w][b],$$

$$\Pi_i[w][b] := \min\nolimits_{\boldsymbol{s_1} \in S_{i-1}(X[w|b-1]) \text{ and } \boldsymbol{s_2} \in S_{k-i}(X[w+b|n-b])} \pi_i(\boldsymbol{s_1} X_{w+b-1} \boldsymbol{s_2}).$$

Recall that $\pi_i(\cdot) := (\psi_i(\cdot), \omega_i(\cdot))$ and ψ_i has a higher priority than ω_i in defining the order π_i. We therefore first calculate the optimal ψ_i, defined as

$$\Psi_i[w][b] := \min\nolimits_{\boldsymbol{s_1} \in S_{i-1}(X[w|b-1]) \text{ and } \boldsymbol{s_2} \in S_{k-i}(X[w+b|n-b])} \psi_i(\boldsymbol{s_1} X_{w+b-1} \boldsymbol{s_2})$$

and then calculate the optimal ω, defined as

$$\Omega_i[w][b] := \min\nolimits_{\boldsymbol{s_1} \in S_{i-1}(X[w|b-1]) \text{ and } \boldsymbol{s_2} \in S_{k-i}(X[w+b|n-b]) \text{ and } \psi_i(\boldsymbol{s_1} X_{w+b-1} \boldsymbol{s_2}) = \Psi_i[w][b]} \omega_i(\boldsymbol{s_1} X_{w+b-1} \boldsymbol{s_2}).$$

We design an $O(d)$ time algorithm to calculate $\Psi_i[w][b]$. Recall the definition of ψ_i and note that of X_{w+b-1} is the pivot, we have

$$\psi_i(\boldsymbol{s_1} X_{w+b-1} \boldsymbol{s_2}) = (\psi_R(\overleftarrow{\boldsymbol{s_1}}) + C_P[i][X_{w+b+1}] + \psi_F(\boldsymbol{s_2})) \bmod d.$$

We first collect values $\psi_R(\overleftarrow{\boldsymbol{s_1}})$ and $\psi_F(\boldsymbol{s_2})$ can take; we use two vectors, V_1 and V_2, to store them. For each $j = 0, 1, \cdots, d-1$, we define $j \in V_1$ if and only if there exists $\overleftarrow{\boldsymbol{s_1}} \in S_{i-1}(X[w|b-1])$ such that $\psi_R(\overleftarrow{\boldsymbol{s_1}}) = j$; similarly, we define $j \in V_2$ if and only if there exists $\boldsymbol{s_2} \in S_{k-i}(X[w+b|n-b])$ such that $\psi_F(\boldsymbol{s_2}) = j$. These two (sorted) vectors can be calculated easily given the solutions of the subproblems in the iterating step. In fact, $j \in V_1$ if and only if $R_{\min}[w][b-1][i-1][j] \neq \text{NaN}$, and $j \in V_2$ if and only if $F_{\min}[w+b][n-b][k-i][j] \neq \text{NaN}$. ($R_{\min}$ can be replaced with $R_{\max}$, and $F_{\min}$ can be replaced with $F_{\max}$ here.) Once V_1 and V_2 are ready, calculating $\Psi_i[w][b]$ becomes:

$$\Psi_i[w][b] = \min_{j_1 \in V_1 \text{ and } j_2 \in V_2}((j_1 + C_P[i][X_{w+b+1}] + j_2) \bmod d).$$

This optimization problem can be solved in $O(d)$ time, assuming that a binary vector of size d can be fit in a machine word, or in $O(d \cdot \log d)$ time for arbitrary d. We leave the algorithmic details in Supplementary Note 2.

As for $\Omega_i[w][b]$, to satisfy that $\psi_i(\boldsymbol{s_1} X_{w+b-1} \boldsymbol{s_2}) = \Psi_i[w][b]$, we enumerate how it gets distributed across the three parts: if we assume that $\psi_F(\overleftarrow{\boldsymbol{s_1}}) = j_1$, then we know $\psi_R(\boldsymbol{s_2}) = (\Psi_i[w][b] - j_1 - C_P[i][X_{w+b-1}] + d) \bmod d$, which we define as j_2. Recall that

$$\omega_i(\boldsymbol{s_1} X_{w+b-1} \boldsymbol{s_2}) = \omega_R(\overleftarrow{\boldsymbol{s_1}}) \cdot B_P[i][X_{w+b-1}]_1 + A_P[i][X_{w+b-1}] + \omega_F(\boldsymbol{s_2}) \cdot B_P[i][X_{w+b-1}]_2.$$

We have

$$\Omega_i[w][b] = \max_{0 \le j_1 < d} \left(\max_{\boldsymbol{s_1} \in S_{i-1}(X[w|b-1]) \text{ and } \psi_R(\boldsymbol{s_1}) = j_1} \omega_R(\overleftarrow{\boldsymbol{s_1}}) \cdot B_P[i][X_{w+b-1}]_1 + A_P[i][X_{w+b-1}] \right.$$
$$\left. + \max_{\boldsymbol{s_2} \in S_{k-i}(X[w+b|n-b]) \text{ and } \psi_F(\boldsymbol{s}) = j_2} \omega_F(\boldsymbol{s}) \cdot B_P[i][X_{w+b-1}]_2 \right).$$

The two optimization problems in it in fact are already solved in the iterating-step: the binary values in $B_P[i][X_{w+b-1}]$ determines whether $F_{\min}/R_{\min}$ or $F_{\max}/R_{\max}$ should be used. To see this, define

$$R_i[w][b][j_1] := \begin{cases} +R_{max}[w][b-1][i-1][j_1], & \text{if } B_P[i][X_{w+b-1}]_1 = +1 \\ -R_{min}[w][b-1][i-1][j_1], & \text{if } B_P[i][X_{w+b-1}]_1 = -1 \end{cases}$$

and

$$F_i[w][b][j_2] := \begin{cases} +F_{max}[w+b][n-b][k-i][j_2], & \text{if } B_P[i][X_{w+b-1}]_2 = +1 \\ -F_{min}[w+b][n-b][k-i][j_2], & \text{if } B_P[i][X_{w+b-1}]_2 = -1 \end{cases}$$

Then $R_i[w][b][j_1]$ and $F_i[w][b][j_2]$ exactly give the optimal solution of the first and third term in the parenthesis. Formally,

$$\Omega_i[w][b] = \max_{0 \le j_1 < d} \left(R_i[w][b][j_1] + A_P[i][X_{w+b-1}] + F_i[w][b][j_2] \right).$$

Calculating $\Omega_i[w][b]$ also takes $O(d)$ time. Combined, calculating $\Pi_i[w][b] = (\Psi_i[w][b], \Omega_i[w][b])$ takes $O(d)$ time for a single pair of w and b. Hence, the entire optimizing-step runs in $O(Nnkd)$ time.

At the end, we combine all these to compute $\Pi_i[w]$ and $\boldsymbol{z}_i^*[w]$. By definitions, we can simply calculate $\Pi_i[w] = \min_{1 \le b \le n} \Pi_i[w][b]$. The optimal subsequence $\boldsymbol{z}_i^*[w]$ can be obtained by traceback. The entire algorithm that finds k seeds for every length-n window in a given length-N sequence therefore also runs in $O(Nnkd)$ time.

Supplementary Note 2: Algorithm for calculating $\Psi_i[w][b]$

Calculating $\Psi_i[w][b]$ goes down to solve this abstracted problem: given integer d, integer d_0, and two sorted lists V_1 and V_2 for which all elements are in $\{0, 1, \cdots, d-1\}$, calculate $\min_{j_1 \in V_1, j_2 \in V_2}(j_1 + d_0 + j_2) \bmod d$. The constant d_0 stands for $C_P[i][X_{w+b+1}]$ in the original task. Below we design two algorithms: the first one runs in $O(d)$ time assuming that a $2d$ bit-vector can be fit in a machine word; the second algorithm runs in $O(d \cdot \log d)$ solves this problem for arbitrarily large d.

Algorithm 1. We first transfer V_1 into a number x_1 which can be stored as a d-bit vector:

$$x_1 := \sum_{j \in V_1} 2^j.$$

We use $x_1[j]$ to represent the j-th bit of its underlying bit-vector, formally defined as $x_1[j] := \lfloor x_1/2^j \rfloor \bmod 2$. Clearly, for any $0 \le j < d$, we have $x_1[j] = 1$ if and only if $j \in V_1$.

We then calculate x, defined below. For each element $j \in V_2$ we shift the bit-vector of x_1 to the left by j bits to get a new bit-vector (of size $2d$ bits, with possible padding 0s to its left). We calculate x by bit-operation OR over all these new bit-vectors. Clearly, x is of size $2d$ bits as well.

$$x := \bigvee_{j \in V_2} x_1 \times 2^j.$$

We now show that, the i-th bit of x equals to 1, i.e., $x[i] = 1$, if and only if there exists $j_1 \in V_1$ and $j_2 \in V_2$, such that $j_1 + j_2 = i$. Considering how x is calculated, we have that $x[i] = 1$ if and only if there must be $j_2 \in V_2$ such that $(x_1 \times 2^{j_2})[i] = 1$. We can continue to write:

$$\begin{aligned} & x[i] = 1 \\ \iff & \exists j_2 \in V_2 \text{ s.t. } (x_1 \times 2^{j_2})[i] = 1 \\ \iff & \exists j_2 \in V_2 \text{ s.t. } x_1[i - j_2] = 1 \\ \iff & \exists j_2 \in V_2 \text{ s.t. } i - j_2 \in V_1 \\ \iff & \exists j_1 \in V_1, j_2 \in V_2 \text{ s.t. } j_1 + j_2 = i. \end{aligned}$$

Now we transform x back to a set V by defining $x[i] = 1$ if and only if $i \in V$. In other words, V now stores all the possible values that can be obtained by $j_1 + j_2$ where $j_1 \in V_1$ and $j_2 \in V_2$. The final solution can be calculated by enumerating elements in V:

$$\min_{j_1 \in V_1, j_2 \in V_2}(j_1 + d_0 + j_2) \bmod d := \min_{i \in V}(i + d_0) \bmod d.$$

In this algorithm, computing x_1 takes $O(d)$ time; calculating x takes $O(d)$ time as well because there are at most d multiplication (bit-shifting) and d bitwise OR operations, which of which can be done in $O(1)$ time. The final minimization takes $O(d)$ time. Therefore, the overall running time of this algorithm is $O(d)$.

Algorithm 2. We have another $O(d \cdot \log d)$ algorithm for general cases. For each value j_1 in V_1, the optimal value from V_2 is $j^* := (2d - j_1 - d_0) \bmod d$, as in this case $(j_1 + j^* + d_0) \bmod d = 0$. But j^* might not exist in V_2. To find the optimal value in V_2, we note that for each $j_2 \in V_2$ the objective function can be represented in a simple closed form using j^*:

$$(j_1 + d_0 + j_2) \bmod d = \begin{cases} j_2 - j^* & \text{if } j^* \le j_2 < d \\ d - j^* + j_2 & \text{if } 0 \le j_2 < j^* \end{cases}$$

This closed form can also be illustrated and verified using the table given below.

j_2	0	1	$\cdots$	$j^* - 1$	j^*	$j^* + 1$	$\cdots$	$d - 1$
$(j_1 + d_0 + j_2) \bmod d$	$d - j^*$	$d - j^* + 1$	$\cdots$	$d - 1$	0	1	$\cdots$	$d - 1 - j^*$

For each j_1, above closed form gives an easy way to find optimal value in V_2: among these j_2 in V_2 satisfying $j^* \leq j_2$, the optimal one (which gives the smallest objective value) would be the smallest such j_2; among these j_2 in V_2 satisfying $j_2 < j^*$, the optimal one (which gives the smallest objective value) would be the smallest such j_2 as well. Note that, for any $j_2 \geq j^*$, it always gives a smaller objective value than any $j_2 < j^*$.

Hence, we can apply binary search on V_2 to find the optimal j_2. Specifically, we search V_2 to find the first value that is equal to or larger than j^*. If it exists, denoting it as p, we calculate the corresponding objective value as $p - j^*$. Otherwise, we find the smallest value in V_2 (which can be done in $O(1)$ time as we assume both V_1 and V_2 are sorted), denoting it as q, we calculate the corresponding objective value as $d - j^* + q$. The overall optimal objective value will be the smallest one by enumerating all $j_1 \in V_1$.

For each $j_1 \in V_1$, above binary search takes $O(\log d)$ time; the entire algorithm runs in $O(d \cdot \log d)$ time.

Supplementary Note 3: SIMD parallelism

We apply SIMD (single instruction, multiple data) parallelism to expedite seed computation. The iterating-step solves $O(Nnkd)$ subproblems in $O(Nnkd)$ time. With SIMD parallelism, we can concurrently compute d subproblems. This reduces the running time of iterating step to $O(Nnk)$. In the optimizing step, getting the optimal value of ψ still takes $O(Nnkd)$ but it only constitutes a tiny portion of the optimizing step and the constant factor of the time complextiy is small. Calculating the optimal value of ω for each seed, which dominates the optimizing step, can also be improved to $O(Nnk)$ with SIMD instructions.

We give the details of SIMD for the iterating-step. Within the recurrences, operations such as addition, multiplication, minimum, and maximum operations can be replaced with SIMD instructions in the CPU intrinsics instruction set. Take the subproblem $F_{\min}[w][b][l][j]$ as an example, we can get all the values of $F_{\min}[w][b][l][j], 0 \leq j < d$ in $O(1)$ time.

$$F_{\min}[w][b][l][j] = \min \begin{cases} F_{\min}[w][b-1][l][j] \\ A_F[l][j][\sigma] \cdot B_F[l][j][\sigma]_2 + \begin{cases} +F_{\min}[w][b-1][l-1][j'], & \text{if } B_F[l][j][\sigma]_1 = +1 \\ -F_{\max}[w][b-1][l-1][j'], & \text{if } B_F[l][j][\sigma]_1 = -1 \end{cases} \end{cases}$$

We first load all the values of $A_F[l][j][\sigma], B_F[l][j][\sigma]_2, , 0 \leq j < d$ into two registers. When iterating j from 0 to $d-1$, the multiplication of $A_F[l][j][\sigma]$ and $B_F[l][j][\sigma]_2$ can be simplified as a single SIMD multiply instruction can directly get all the results. Besides, we can transform $B_F[l][j][\sigma]_1$ into single mask number(an integer) where each bit represents the selection of $+F_{\min}[w][b-1][l-1][j']$ or $-F_{\max}[w][b-1][l-1][j']$. Similarly addition, maximum and minimum operations can also be replaced by SIMD instructions. In summary, transforming all operations in the recurrence into SIMD instructions reduces the time complexity of the entire iterating-steps to $O(Nnk)$.

In the optimizing-step, calculating $\Omega_i[w][b]$ can also benefit from similar SIMD instructions which results in $O(1)$ time. Although $\Psi_[w][b]$ still requires $O(d)$ running time, it is notably faster comparing to iterating-step and calculating $\Omega_i[w][b]$. Consequently, SIMD instructions contribute significantly to saving time in the overall seed calculation.

In detail, we require that d can not exceed 32 which allows to save at most 32 different 16-bits values in a 512-bits register. Regardless of the value of d, the overall running time remains nearly constant.

Supplementary Note 4: ABCk tables used by SubseqHash2r

Here we derive the required property on the ABCk tables in order to generate the same set of seeds for a string and its reverse complement. Let $\boldsymbol{z} = z_1 z_2 \cdots z_k$ and therefore $\bar{\boldsymbol{z}} = \overline{z_k}\,\overline{z_{k-1}} \cdots \overline{z_1}$, where we use $\overline{z_k}$ to represent the "complement" of letter z_k. We require $\pi_i(\boldsymbol{z}) = \pi_{k-i+1}(\bar{\boldsymbol{z}})$, for every $1 \leq i \leq k$. Recall that $\pi_{k-i+1}(\bar{\boldsymbol{z}}) = (\psi_{k-i+1}(\bar{\boldsymbol{z}}), \omega_{k-i+1}(\bar{\boldsymbol{z}}))$ and

$$\psi_i(\boldsymbol{z}) = (\psi_R(z_{i-1} z_{i-2} \cdots z_1) + C_P[i][z_i] + \psi_F(z_{i+1} z_{i+2} \cdots z_k)) \bmod d,$$
$$\psi_{k-i+1}(\bar{\boldsymbol{z}}) = (\psi_R(\overline{z_{i+1}}\,\overline{z_{i+2}} \cdots \overline{z_k}) + C_P[k-i+1][\overline{z_i}] + \psi_F(\overline{z_{i-1}}\,\overline{z_{i-2}} \cdots \overline{z_1})) \bmod d.$$

In order to make $\psi_{k-i+1}(\bar{\boldsymbol{z}}) = \psi_i(\boldsymbol{z})$ we require the following

$$C_P[k-i+1][\overline{z_i}] = C_P[i][z_i]$$
$$\psi_R(\overline{z_{i+i}}\,\overline{z_{i+2}} \cdots \overline{z_k}) = \psi_F(z_{i+1} z_{i+2} \cdots z_k)$$
$$\psi_F(\overline{z_{i-1}}\,\overline{z_{i-2}} \cdots \overline{z_1}) = \psi_R(z_{i-1} z_{i-2} \cdots z_1)$$

By the definitions of ψ_F and ψ_R, they lead to the following for every $1 \leq i \leq k$ and $z \in \Sigma$.

$$C_P[k-i+1][z] = C_P[i][\bar{z}]$$
$$C_F[i][z] = C_R[i][\bar{z}]$$

The same approach can be used to derive the requirements needed for making $\omega_{k-i+1}(\bar{\boldsymbol{z}}) = \omega_i(\boldsymbol{z})$, which we directly show below, for every $1 \leq i \leq k$, $z \in \Sigma$, and $0 \leq j < d$:

$$A_P[k-i+1][z] = A_P[i][\bar{z}]$$
$$B_P[k-i+1][z]_1 = B_P[i][\bar{z}]_2$$
$$A_F[i][j][z] = A_R[i][j][\bar{z}]$$
$$B_F[i][j][z]_1 = B_R[i][j][\bar{z}]_1$$
$$B_F[i][j][z]_2 = B_R[i][j][\bar{z}]_2$$

Supplementary Note 5: An example for the ABCk order

We give an example for the ABCk order, determined by the three random tables shown in Supplementary Table 1, 2, and 3 with $k = 6$, $d = 5$, and $\Sigma = \{A, C, G, T\}$. The score of $\boldsymbol{z}$ = CTAACT can be calculated according to the tables following the recurrences, with detailed given below.

We first compute ψ_F, ω_F using tables A_F, B_F, and C_F, and ψ_R, ω_R with tables A_R, B_R, and C_R. The results are shown in the table below.

	Reverse	Pivot	Forward	ψ_R	ω_R	ψ_F	ω_F
π_1	$\varnothing$	C	TAACT	0	0	3	-155
π_2	C	T	AACT	3	-18	3	-52
π_3	TC	A	ACT	0	-37	2	-156
π_4	ATC	A	CT	4	1	2	-36
π_5	AATC	C	T	0	9	0	79
π_6	CAATC	T	$\varnothing$	2	-191	0	0

Now we can calculate the values of k score functions π_1, π_2, $\cdots$, π_6 for $\boldsymbol{Z}$ = CTAACT based on ψ_F, ω_F, ψ_R, ω_R, and table A_P, B_P, C_P.

$$\psi_1(\text{CTAACT}) = (\phi_R(\varnothing) + C_P[1][\text{C}] + \phi_F(\text{TAACT})) \bmod 5 = (0 + 1 + 3) \bmod 5 = 4,$$
$$\omega_1(\text{CTAACT}) = \omega_R(\varnothing) \cdot B_P[1][\text{C}]_1 + A_P[1][\text{C}] + \omega_F(\text{TAACT}) \cdot B_P[1][\text{C}]_2 = 0 * (-1) + 82 + (-155) * (-1) = 237.$$

$$\psi_2(\text{CTAACT}) = (\phi_R(\text{C}) + C_P[2][\text{T}] + \phi_F(\text{AACT})) \bmod 5 = (3 + 0 + 3) \bmod 5 = 1,$$
$$\omega_2(\text{CTAACT}) = \omega_R(\text{C}) \cdot B_P[2][\text{T}]_1 + A_P[2][\text{T}] + \omega_F(\text{AACT}) \cdot B_P[2][\text{T}]_2 = -18 * (+1) + (-95) + (-52) * (+1) = -165.$$

$$\psi_3(\text{CTAACT}) = (\phi_R(\text{TC}) + C_P[3][\text{A}] + \phi_F(\text{ACT})) \bmod 5 = (0 + 3 + 2) \bmod 5 = 0,$$
$$\omega_3(\text{CTAACT}) = \omega_R(\text{TC}) \cdot B_P[3][\text{A}]_1 + A_P[3][\text{A}] + \omega_F(\text{ACT}) \cdot B_P[3][\text{A}]_2 = -37 * (-1) + (-12) + (-156) * (+1) = -131.$$

$$\psi_4(\text{CTAACT}) = (\phi_R(\text{ATC}) + C_P[4][\text{A}] + \phi_F(\text{CT})) \bmod 5 = (4 + 0 + 2) \bmod 5 = 1,$$
$$\omega_4(\text{CTAACT}) = \omega_R(\text{ATC}) \cdot B_P[4][\text{A}]_1 + A_P[4][\text{A}] + \omega_F(\text{CT}) \cdot B_P[4][\text{A}]_2 = 1 * (-1) + 49 + (-36) * (-1) = 84.$$

$$\psi_5(\text{CTAACT}) = (\phi_R(\text{AATC}) + C_P[5][\text{C}] + \phi_F(\text{T})) \bmod 5 = (0 + 1 + 0) \bmod 5 = 1,$$
$$\omega_5(\text{CTAACT}) = \omega_R(\text{AATC}) \cdot B_P[5][\text{C}]_1 + A_P[5][\text{C}] + \omega_F(\text{T}) \cdot B_P[5][\text{C}]_2 = 9 * (+1) + (-36) + 79 * (-1) = -106.$$

$$\psi_6(\text{CTAACT}) = (\phi_R(\text{CAATC}) + C_P[6][\text{T}] + \phi_F(\varnothing)) \bmod 5 = (2 + 2 + 0) \bmod 5 = 4,$$
$$\omega_6(\text{CTAACT}) = \omega_R(\text{CAATC}) \cdot B_P[6][\text{T}]_1 + A_P[6][\text{T}] + \omega_F(\varnothing) \cdot B_P[6][\text{T}]_2 = -191 * (-1) + 16 + 0 * (+1) = 207.$$

The k scores of $\boldsymbol{z}$ = CTAACT are listed below. Similarly, we also get the scores of $\boldsymbol{z'}$ = CCAACT.

$\pi_1(\text{CTAACT}) = (4, 237)$ $\pi_1(\text{CCAACT}) = (1, -25)$
$\pi_2(\text{CTAACT}) = (1, -165)$ $\pi_2(\text{CCAACT}) = (2, 155)$
$\pi_3(\text{CTAACT}) = (0, -131)$ $\pi_3(\text{CCAACT}) = (1, -151)$
$\pi_4(\text{CTAACT}) = (1, 84)$ $\pi_4(\text{CCAACT}) = (2, 119)$
$\pi_5(\text{CTAACT}) = (1, -106)$ $\pi_5(\text{CCAACT}) = (3, -21)$
$\pi_6(\text{CTAACT}) = (4, 207)$ $\pi_6(\text{CCAACT}) = (2, 186)$

Note that the edit distance between $\boldsymbol{z}$ and $\boldsymbol{z'}$ is 1, but all the k scores are drastically changed. This is a desired property and is the goal of the design of the ABCk orders. Because of this, each of the k orders in the ABCk order behaves similarly to a pure random order, so that the probability of hash collision between two strings can be approximately the Jaccard index between the two sets of length-k subsequences.

Supplementary Table 1: An example of random tables A_F, B_F, C_F used in an ABCk order. Entries in table A_F are integers drawn from $[10, 100]$.

(a) Table A_F

		σ			
i	j	A	C	G	T
1	0	52	87	27	79
	1	46	40	52	70
	2	56	36	82	91
	3	99	22	52	23
	4	99	87	50	29
2	0	79	97	46	19
	1	57	49	65	87
	2	70	57	39	72
	3	56	32	58	70
	4	62	96	42	38
3	0	98	15	36	34
	1	14	89	62	39
	2	65	32	96	25
	3	21	84	40	97
	4	17	83	37	70
4	0	23	14	96	60
	1	98	46	74	31
	2	72	96	77	79
	3	41	20	89	99
	4	38	80	95	82
5	0	70	44	37	87
	1	78	96	79	32
	2	98	10	30	98
	3	92	10	58	97
	4	61	94	24	35
6	0	84	34	16	30
	1	48	62	82	43
	2	69	84	92	51
	3	94	65	95	52
	4	87	32	89	44

(b) Table B_F

		σ			
i	j	A	C	G	T
1	0	$(1,-1)$	$(-1,1)$	$(-1,-1)$	$(1,1)$
	1	$(1,-1)$	$(-1,1)$	$(-1,-1)$	$(1,1)$
	2	$(1,-1)$	$(-1,-1)$	$(-1,1)$	$(1,1)$
	3	$(1,-1)$	$(1,1)$	$(-1,1)$	$(-1,-1)$
	4	$(1,-1)$	$(-1,1)$	$(1,1)$	$(-1,-1)$
2	0	$(-1,-1)$	$(-1,1)$	$(1,1)$	$(1,-1)$
	1	$(-1,-1)$	$(1,1)$	$(-1,1)$	$(1,-1)$
	2	$(-1,1)$	$(1,1)$	$(1,-1)$	$(-1,-1)$
	3	$(1,1)$	$(1,-1)$	$(-1,1)$	$(-1,-1)$
	4	$(-1,1)$	$(-1,-1)$	$(1,1)$	$(1,-1)$
3	0	$(1,-1)$	$(1,1)$	$(-1,-1)$	$(-1,1)$
	1	$(-1,-1)$	$(-1,1)$	$(1,-1)$	$(1,1)$
	2	$(-1,-1)$	$(-1,1)$	$(1,1)$	$(1,-1)$
	3	$(1,1)$	$(-1,-1)$	$(-1,1)$	$(1,-1)$
	4	$(1,1)$	$(-1,1)$	$(1,-1)$	$(-1,-1)$
4	0	$(1,-1)$	$(-1,-1)$	$(1,1)$	$(-1,1)$
	1	$(1,-1)$	$(-1,-1)$	$(-1,1)$	$(1,1)$
	2	$(-1,1)$	$(1,-1)$	$(1,1)$	$(-1,-1)$
	3	$(-1,1)$	$(-1,-1)$	$(1,1)$	$(1,-1)$
	4	$(1,-1)$	$(1,1)$	$(-1,-1)$	$(-1,1)$
5	0	$(1,-1)$	$(-1,-1)$	$(1,1)$	$(-1,1)$
	1	$(-1,1)$	$(1,-1)$	$(1,1)$	$(-1,-1)$
	2	$(1,-1)$	$(1,1)$	$(-1,1)$	$(-1,-1)$
	3	$(1,1)$	$(-1,1)$	$(-1,-1)$	$(1,-1)$
	4	$(1,-1)$	$(-1,1)$	$(1,1)$	$(-1,-1)$
6	0	$(-1,-1)$	$(1,1)$	$(-1,1)$	$(1,-1)$
	1	$(1,1)$	$(-1,1)$	$(1,-1)$	$(-1,-1)$
	2	$(1,1)$	$(-1,1)$	$(-1,-1)$	$(1,-1)$
	3	$(-1,1)$	$(1,-1)$	$(1,1)$	$(-1,-1)$
	4	$(-1,-1)$	$(1,1)$	$(1,-1)$	$(-1,1)$

(c) Table C_F

	σ			
i	A	C	G	T
1	4	2	3	0
2	2	4	3	0
3	1	0	2	4
4	1	3	0	2
5	1	0	4	2
6	1	3	2	4

Supplementary Table 2: An example of random tables A_R, B_R, C_R used in an ABCk order. Entries in table A_R are integers drawn from $[10, 100]$.

(a) Table A_R

		σ			
i	j	A	C	G	T
	0	15	44	68	42
	1	28	47	71	79
1	2	48	48	88	14
	3	64	18	99	60
	4	11	92	74	96
	0	15	23	92	33
	1	96	35	89	87
2	2	12	67	21	83
	3	43	29	85	99
	4	39	18	14	70
	0	37	48	60	99
	1	84	24	98	19
3	2	51	23	35	79
	3	66	91	91	26
	4	29	67	93	31
	0	46	46	91	94
	1	14	49	73	31
4	2	49	94	16	44
	3	48	71	66	62
	4	35	12	67	35
	0	93	38	65	86
	1	96	66	91	80
5	2	91	42	33	72
	3	11	41	45	45
	4	60	23	29	24
	0	79	95	17	44
	1	26	63	65	23
6	2	77	36	71	48
	3	29	91	49	63
	4	99	31	92	71

(b) Table B_R

		σ			
i	j	A	C	G	T
	0	$(-1,-1)$	$(1,-1)$	$(-1,1)$	$(1,1)$
	1	$(-1,-1)$	$(1,-1)$	$(1,1)$	$(-1,1)$
1	2	$(-1,-1)$	$(1,-1)$	$(1,1)$	$(-1,1)$
	3	$(-1,-1)$	$(1,-1)$	$(1,1)$	$(-1,1)$
	4	$(-1,-1)$	$(1,-1)$	$(-1,1)$	$(1,1)$
	0	$(1,-1)$	$(-1,-1)$	$(-1,1)$	$(1,1)$
	1	$(1,1)$	$(-1,-1)$	$(1,-1)$	$(-1,1)$
2	2	$(1,-1)$	$(-1,1)$	$(-1,-1)$	$(1,1)$
	3	$(-1,-1)$	$(1,1)$	$(-1,1)$	$(1,-1)$
	4	$(-1,-1)$	$(1,-1)$	$(1,1)$	$(-1,1)$
	0	$(1,1)$	$(1,-1)$	$(-1,-1)$	$(-1,1)$
	1	$(1,-1)$	$(-1,-1)$	$(1,1)$	$(-1,1)$
3	2	$(-1,1)$	$(1,1)$	$(-1,-1)$	$(1,-1)$
	3	$(1,-1)$	$(1,1)$	$(-1,-1)$	$(-1,1)$
	4	$(-1,-1)$	$(1,-1)$	$(1,1)$	$(-1,1)$
	0	$(-1,-1)$	$(1,-1)$	$(-1,1)$	$(1,1)$
	1	$(1,1)$	$(-1,1)$	$(1,-1)$	$(-1,-1)$
4	2	$(1,1)$	$(-1,1)$	$(-1,-1)$	$(1,-1)$
	3	$(1,1)$	$(1,-1)$	$(-1,-1)$	$(-1,1)$
	4	$(1,1)$	$(-1,1)$	$(-1,-1)$	$(1,-1)$
	0	$(-1,-1)$	$(-1,1)$	$(1,1)$	$(1,-1)$
	1	$(1,1)$	$(-1,1)$	$(1,-1)$	$(-1,-1)$
5	2	$(-1,1)$	$(1,-1)$	$(1,1)$	$(-1,-1)$
	3	$(-1,-1)$	$(1,1)$	$(1,-1)$	$(-1,1)$
	4	$(-1,-1)$	$(1,-1)$	$(1,1)$	$(-1,1)$
	0	$(-1,1)$	$(-1,-1)$	$(1,1)$	$(1,-1)$
	1	$(-1,1)$	$(-1,-1)$	$(1,1)$	$(1,-1)$
6	2	$(-1,-1)$	$(1,1)$	$(-1,1)$	$(1,-1)$
	3	$(1,-1)$	$(-1,-1)$	$(1,1)$	$(-1,1)$
	4	$(1,1)$	$(-1,1)$	$(1,-1)$	$(-1,-1)$

(c) Table C_R

	σ			
i	A	C	G	T
1	0	3	1	2
2	4	3	1	2
3	4	2	1	0
4	2	1	4	3
5	0	3	4	2
6	3	1	0	2

Supplementary Table 3: An example of random tables A_P, B_P, C_P used in an ABCk order. Entries in table A_P are integers drawn from $[-100, -10] \cup [10, 100]$.

(a) Table A_P

	σ			
i	A	C	G	T
1	-24	82	-34	11
2	-90	85	33	-95
3	-12	89	29	-12
4	49	71	-73	-18
5	-84	-36	91	70
6	49	-17	-32	16

(b) Table B_P

	σ			
i	A	C	G	T
1	$(-1, 1)$	$(-1, -1)$	$(1, 1)$	$(1, -1)$
2	$(-1, 1)$	$(-1, -1)$	$(1, -1)$	$(1, 1)$
3	$(-1, 1)$	$(1, -1)$	$(-1, -1)$	$(1, 1)$
4	$(-1, -1)$	$(1, 1)$	$(-1, 1)$	$(1, -1)$
5	$(1, 1)$	$(1, -1)$	$(-1, 1)$	$(-1, -1)$
6	$(1, -1)$	$(-1, -1)$	$(1, 1)$	$(-1, 1)$

(c) Table C_P

	σ			
i	A	C	G	T
1	3	1	4	2
2	3	1	4	0
3	3	1	4	0
4	0	2	4	3
5	3	1	4	2
6	4	3	0	2

Supplementary Note 6: Parameters for overlap detection

In the overlap detection experiments of Section 3.5, the following parameters are depicted in Fig. 6 and Supplementary Figures 14–15. For minimizers with window sizes $n = 30, 35$, each curve is drawn with six seed lengths k evenly spaced between 10 and n (when $k = n$, all k-mers are in the seed set). For syncmers with seed lengths $k = 18, 22, 25, 30$, each curve is draw with five s-mer lengths evenly spaced between 2 and k (when $s = k$, all k-mers are selected). For SubseqHash, SubseqHash2r, strobemer, and SubseqHash2w, we choose the window size $n = 60$. For SubseqHash and SubseqHash2r, the seed lengths $k = \lfloor rn \rfloor$ for $r = 0.65, 0.7, 0.75, 0.8, 0.85, 0.9, 0.95$ are plotted.For strobemer, a seed consists of two k-mers for $k = \lfloor rn/2 \rfloor$ with $r = 0.35, 0.45, 0.55, 0.65, 0.75, 0.85, 0.95$, where the first k-mer is the leading k-mer in the window, and the second k-mer is selected by the randstrobe algorithm from the remaining part of the window. For SubseqHash2w, seeds of lengths $k = \lfloor rn \rfloor$ for $r = 0.65, 0.7, 0.75, 0.8, 0.85, 0.9, 0.95$ are depicted; a seed of length k consists of the leading $k_0 = \lfloor k/3 \rfloor$-mer in the window and a SubseqHash2 seed of length $\lceil 2k/3 \rceil$ from the remaining part of the window.

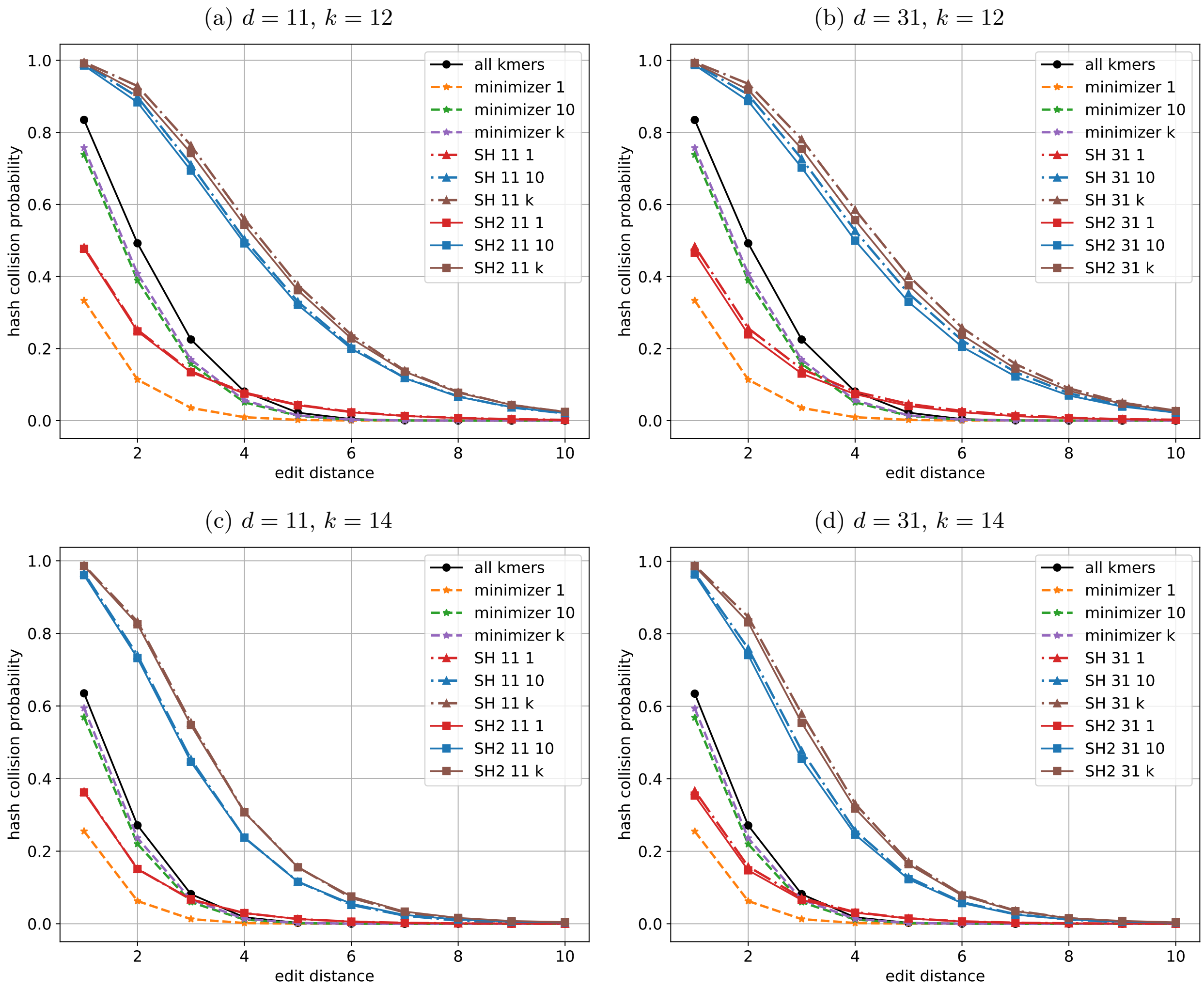

Supplementary Figure 2: The probability of hash collision, estimated using simulations, for different seeding methods with $n = 20$ and $k = 12, 14$.

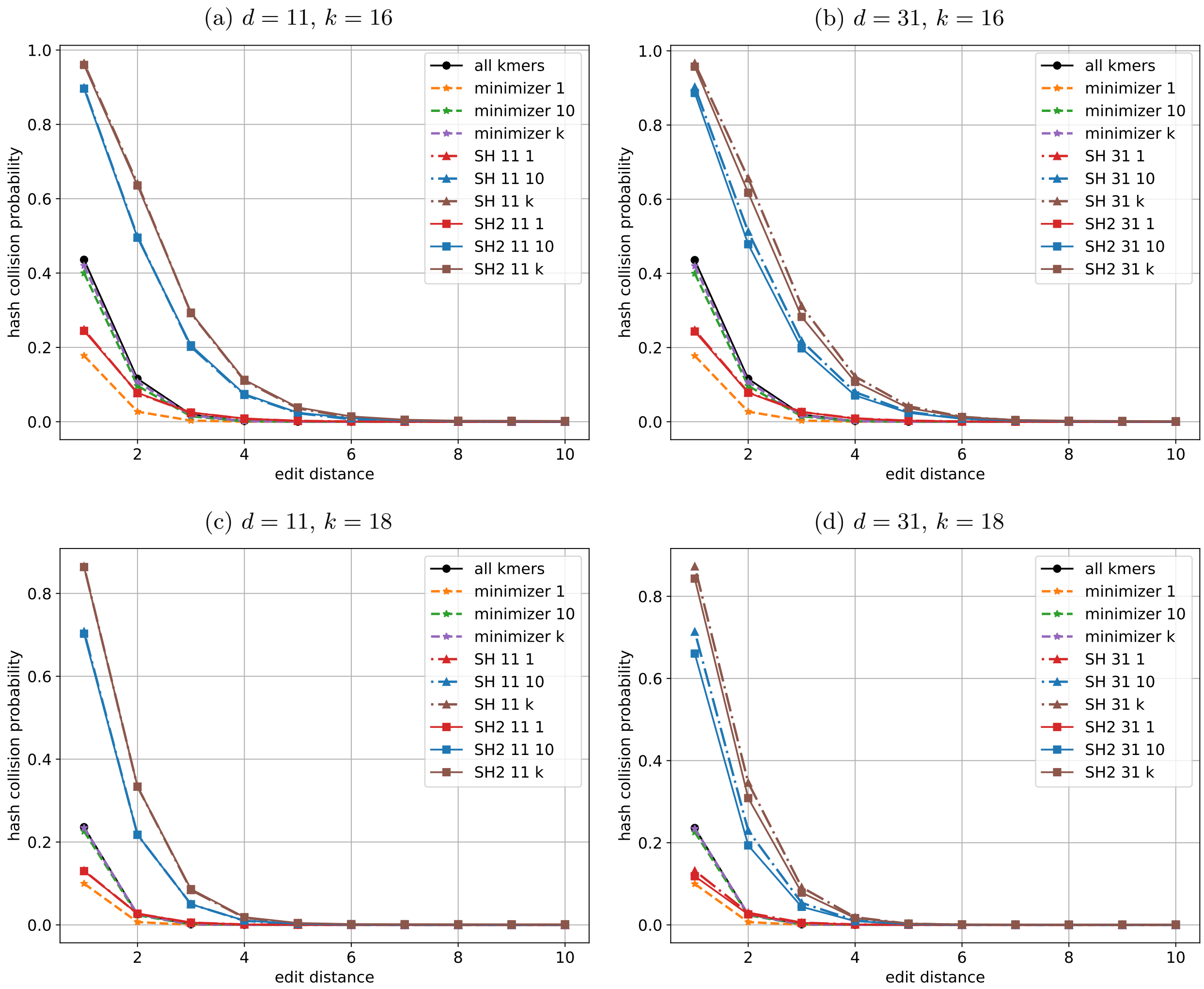

Supplementary Figure 3: The probability of hash collision, estimated using simulations, for different seeding methods with $n = 20$ and $k = 16$, 18.

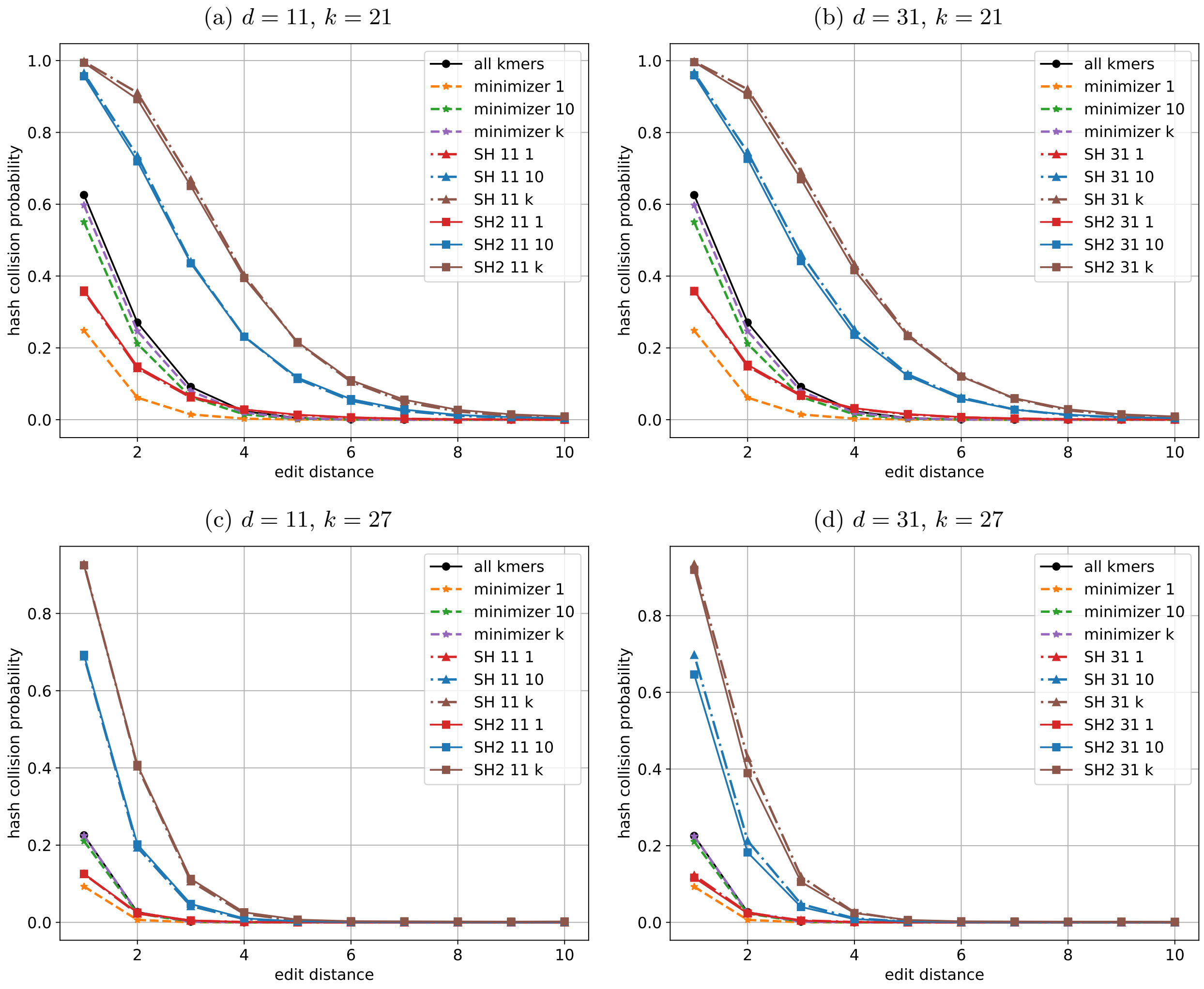

Supplementary Figure 4: The probability of hash collision, estimated using simulations, for different seeding methods with $n = 30$ and $k = 21, 27$.

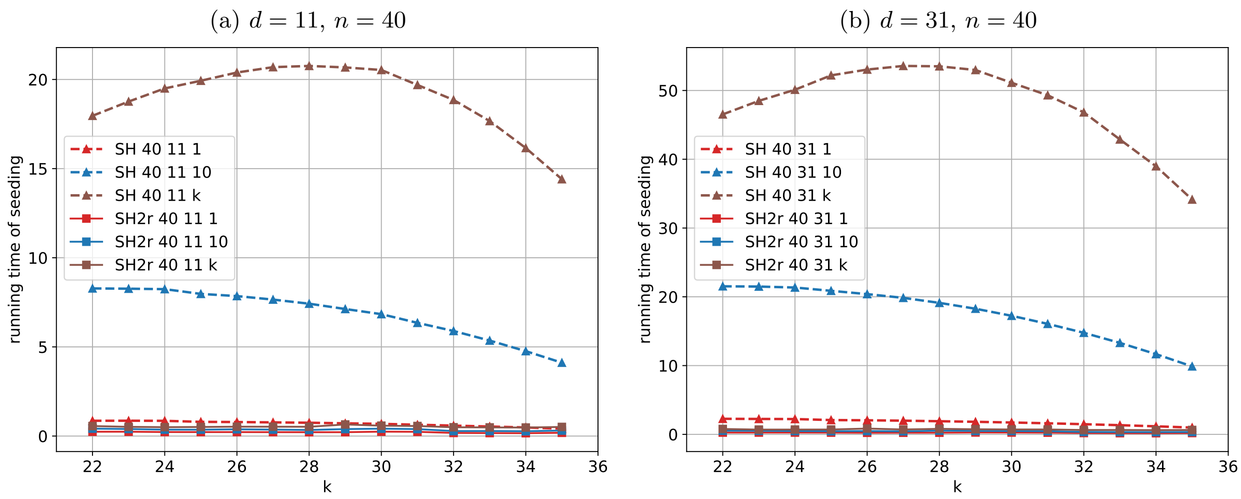

Supplementary Figure 5: The average CPU time (second) of SubseqHash and SubseqHash2 per read with $n = 40$.

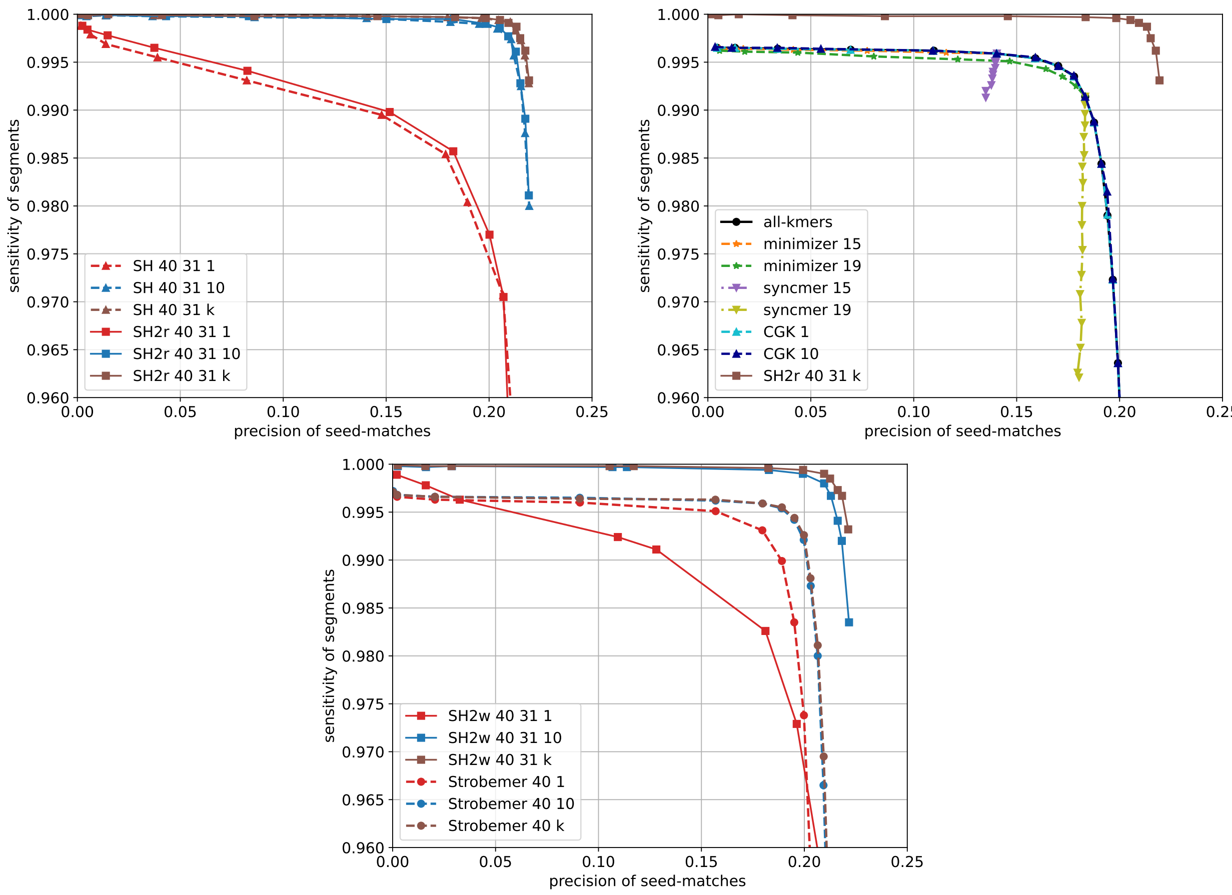

Supplementary Figure 6: Comparison of average seed-match precision and segment sensitivity using a simulated dataset modeled on the sequencing error profile of the Oxford Nanopore dataset SRX27559270 with $n = 40$.

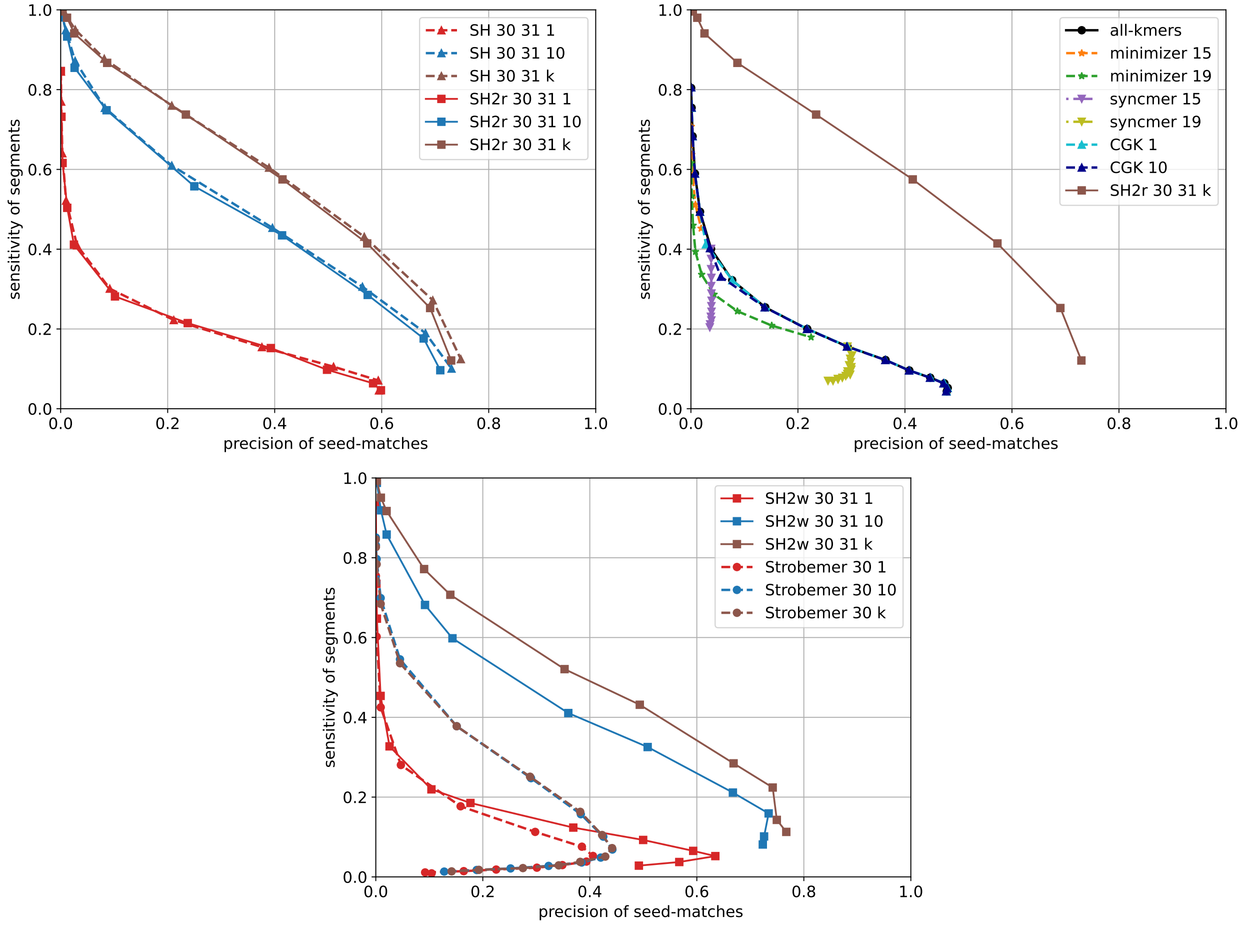

Supplementary Figure 7: Comparison of average seed-match precision and segment sensitivity using a simulated dataset modeled on the sequencing error profile of the PacBio dataset SRX499318 with $n = 30$.

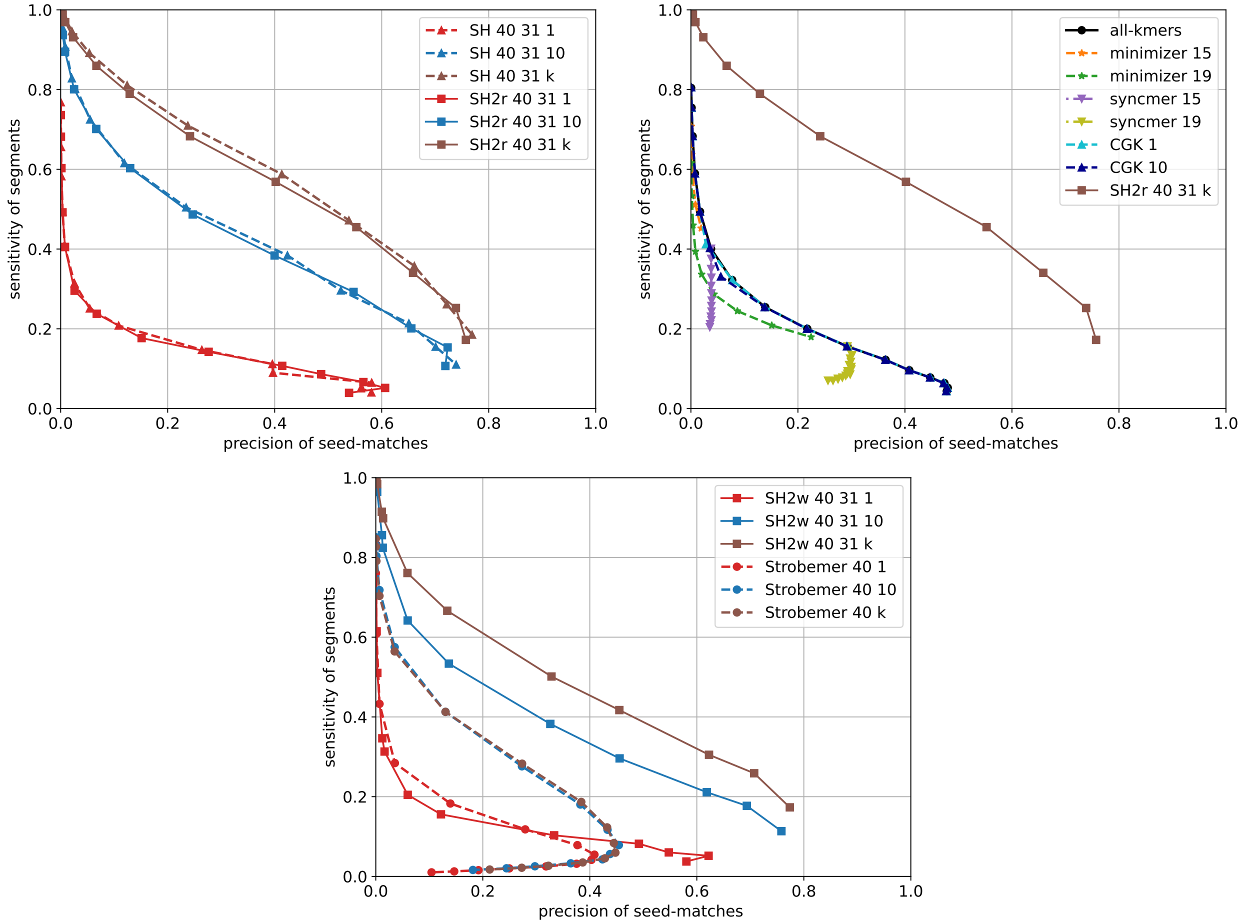

Supplementary Figure 8: Comparison of average seed-match precision and segment sensitivity using a simulated dataset modeled on the sequencing error profile of the PacBio dataset SRX499318 with $n = 40$.

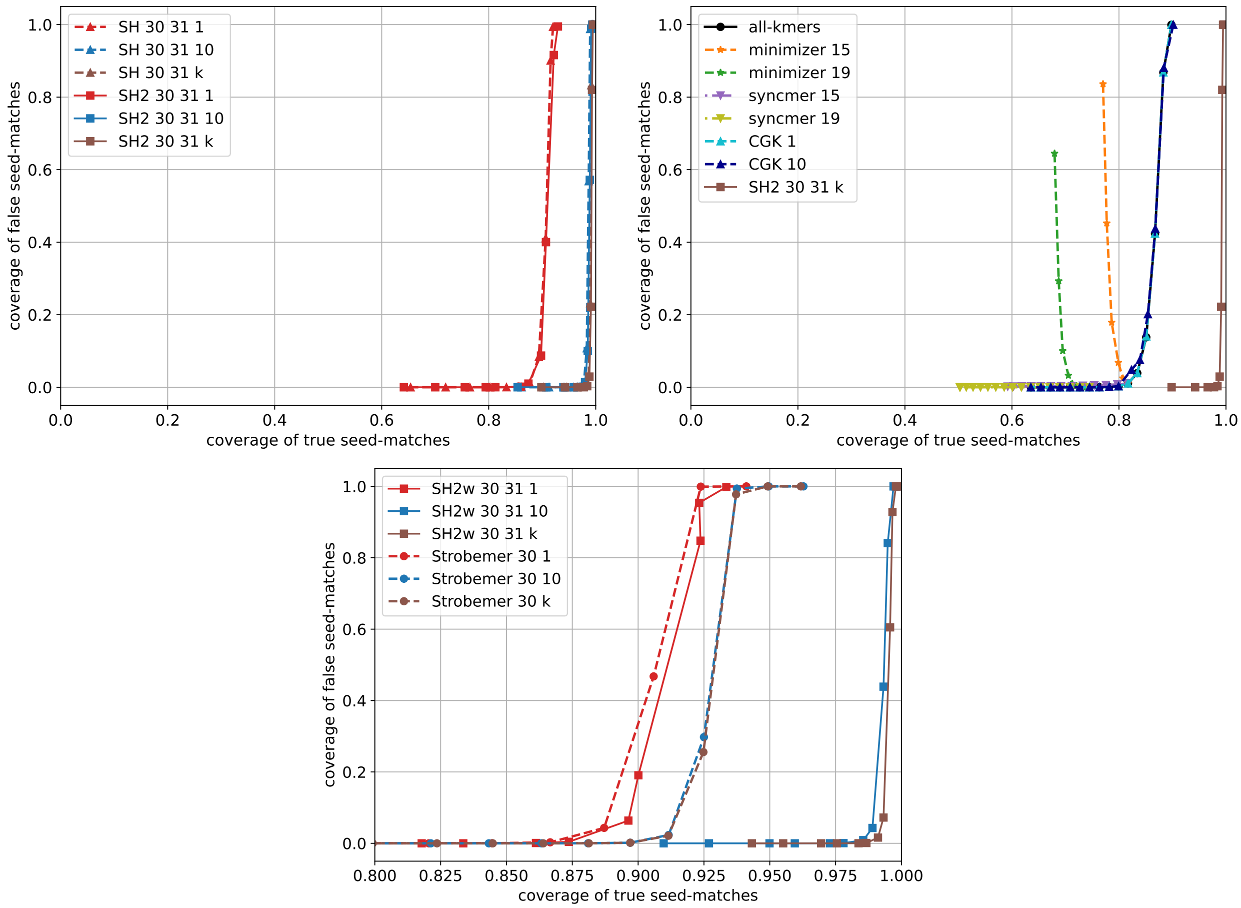

Supplementary Figure 9: The true and false coverage of different seeding methods in sequence alignment with error rate $r = 5\%$, $n = 30$.

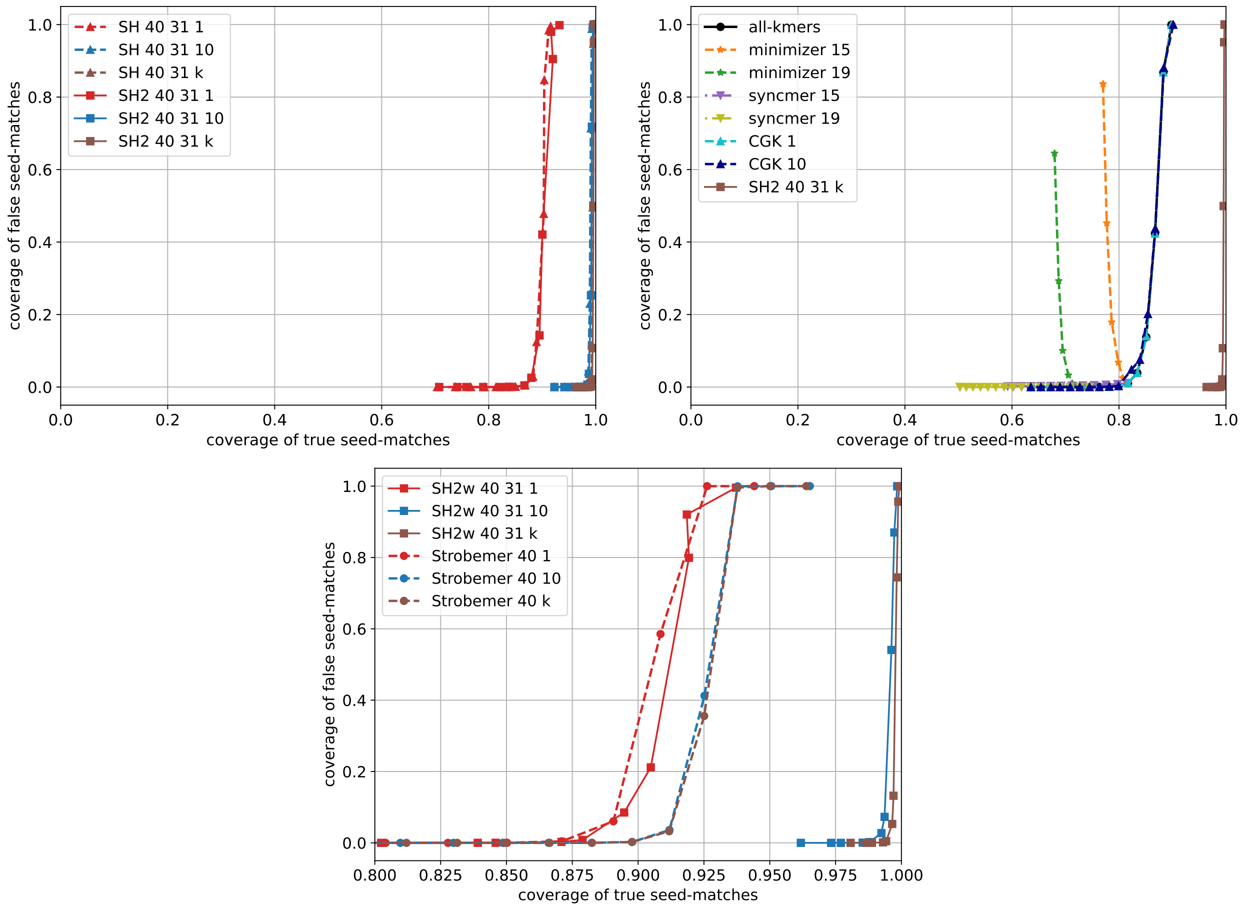

Supplementary Figure 10: The true and false coverage of different seeding methods in sequence alignment with error rate $r = 5\%$, $n = 40$.

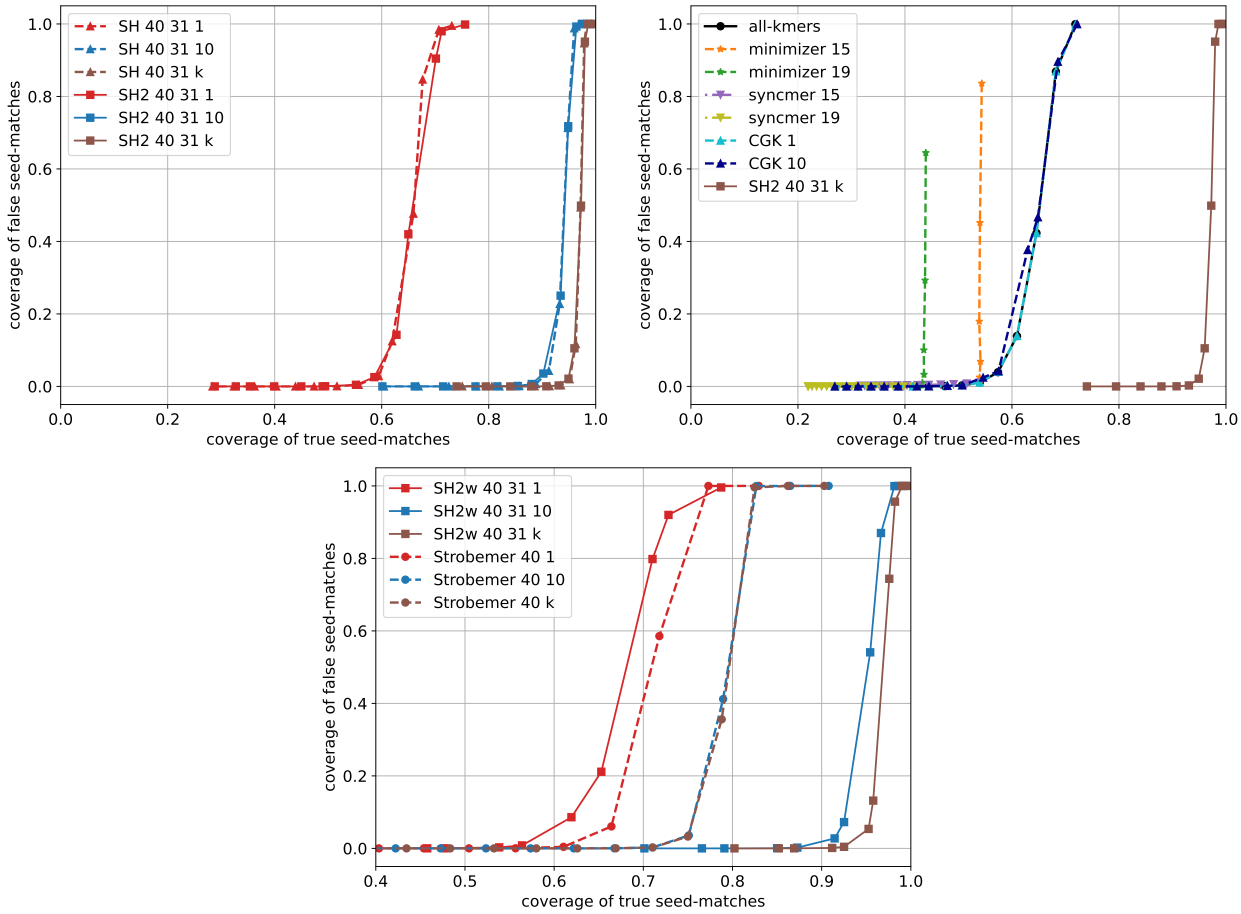

Supplementary Figure 11: The true and false coverage of different seeding methods in sequence alignment with error rate $r = 10\%$, $n = 40$.

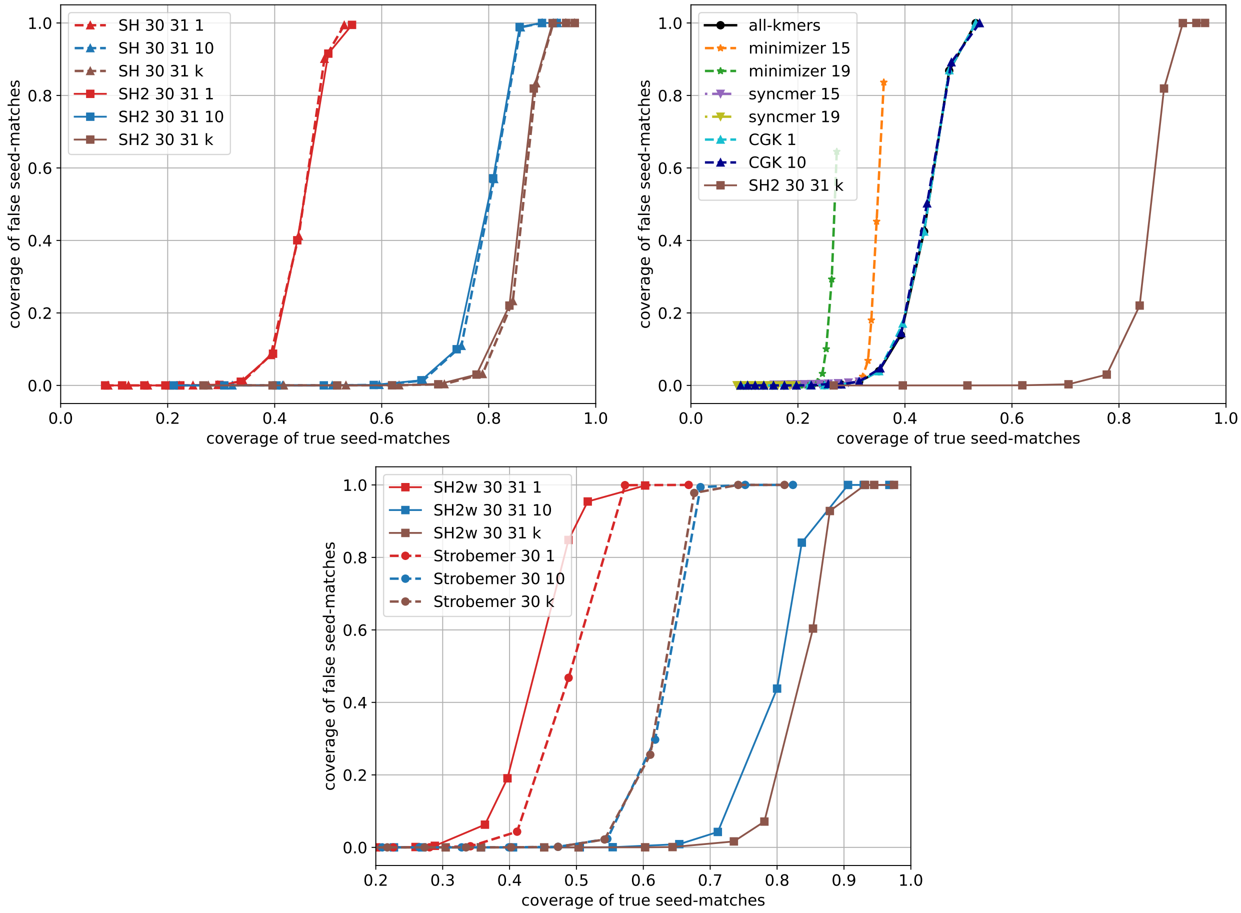

Supplementary Figure 12: The true and false coverage of different seeding methods in sequence alignment with error rate $r = 15\%$, $n = 30$.

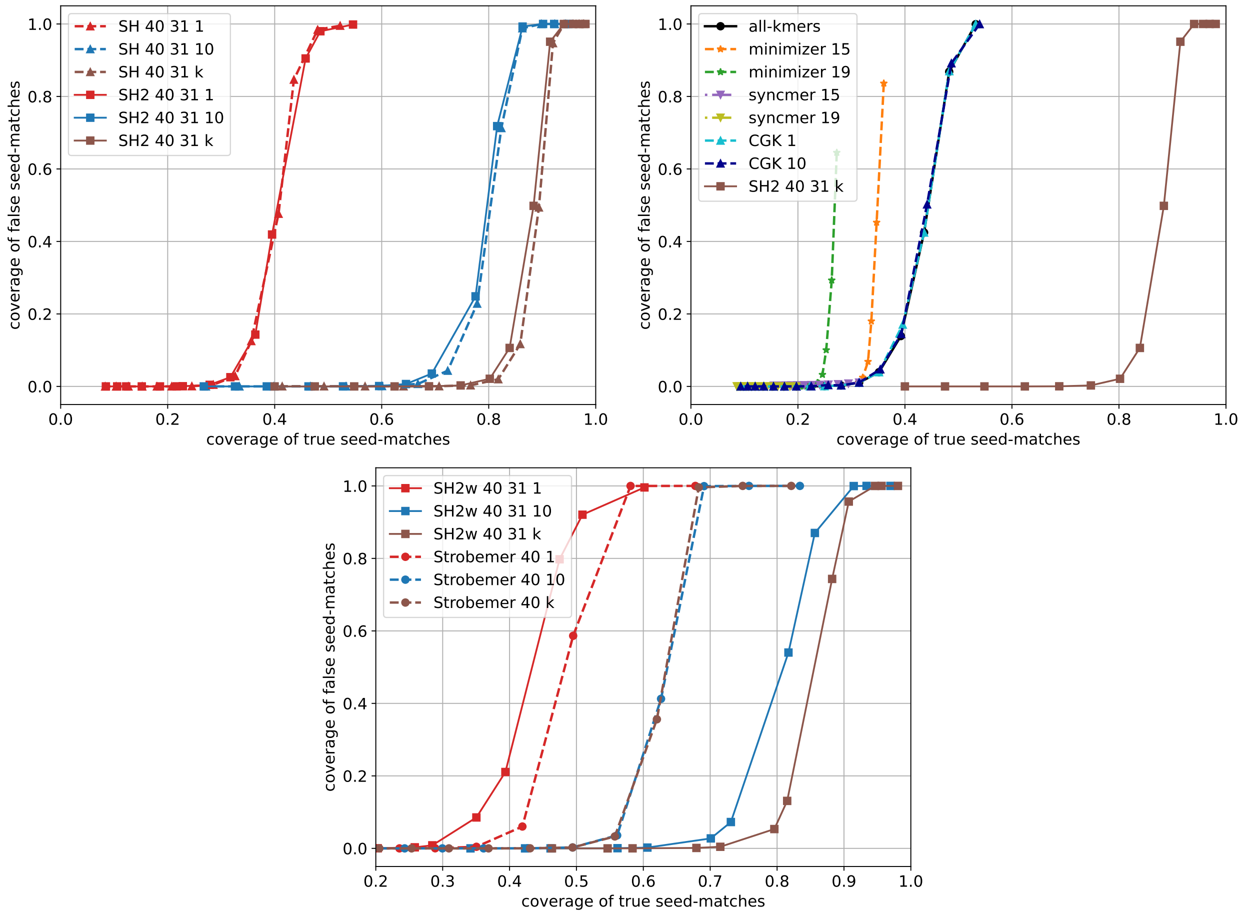

Supplementary Figure 13: The true and false coverage of different seeding methods in sequence alignment with error rate $r = 15\%$, $n = 40$.

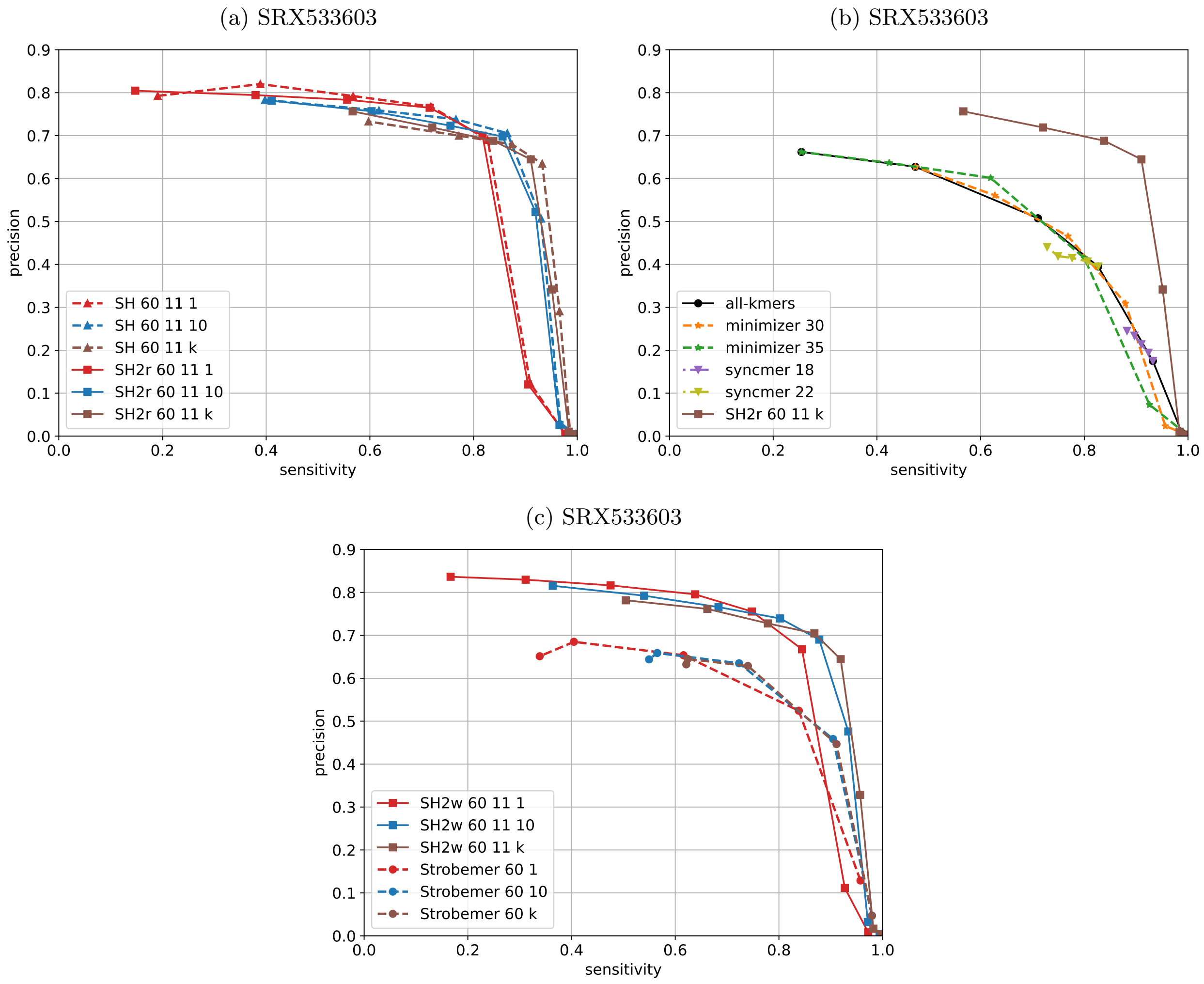

Supplementary Figure 14: Overlap detection results on 10,000 reads sampled from the the *E. coli* SRX533603 dataset with $n = 60$. Figure (a) shows SubseqHash2r achieves similar seed quality as SubseqHash. Figure (b) shows SubseqHash2r produces better seeds than all-kmers, minimizer, and syncmer. Figure (c) shows SubseqHash2w produces better seeds than Strobemer.

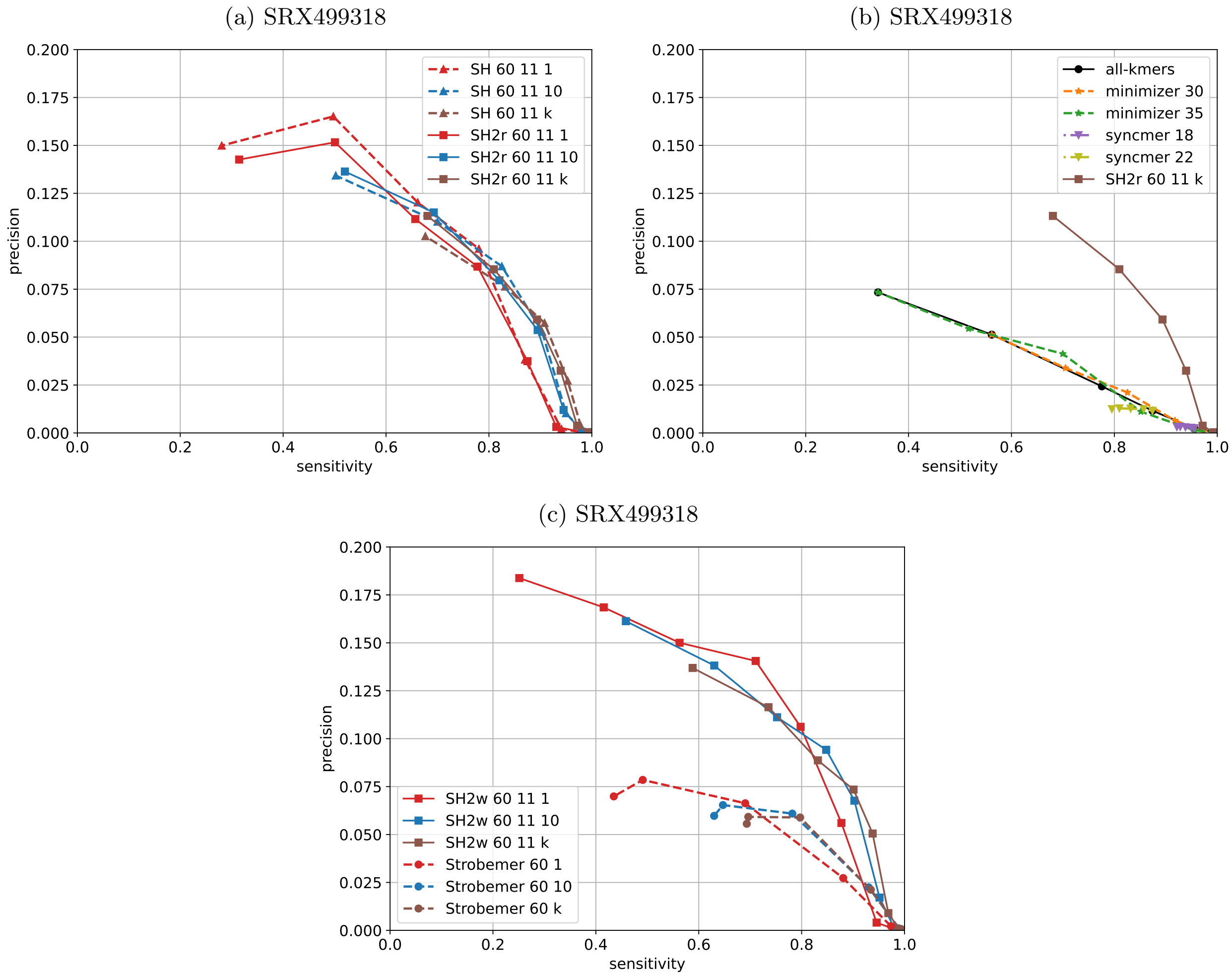

Supplementary Figure 15: Overlap detection results on 10,000 reads sampled from the the *D. melanogaster* SRX499318 dataset with $n = 60$. Figure (a) shows SubseqHash2r achieves similar seed quality as SubseqHash. Figure (b) shows SubseqHash2r produces better seeds than all-kmers, minimizer, and syncmer. Figure (c) shows SubseqHash2w produces better seeds than Strobemer.

Supplementary Table 4: The peak memory usage (MB) of each method in seed generation on a recent Oxford Nanopore long read dataset (SRX27559270).

all-kmers	minimizer	syncmer	CGK-embedding	Strobemer	SubseqHash	SubseqHash2
8.4	8.4	5.9	6.7	7.4	9	455.5
